# Supplementary material for: Understanding the uptake of new hip replacement implants in the UK: a cohort study using data from the National Joint Registry for England and Wales
Source: BMJ Open. 2019 Nov 25;9(11):e029572. doi: 10.1136/bmjopen-2019-029572 (PMC6886938; doi:10.1136/bmjopen-2019-029572)
Supplement: Supplementary data [file bmjopen-2019-029572supp001.pdf]

Supplementary material

Table S1 Uptake of new cups first used between January 1st 2008 and 26th February 2017

Cup/shell brand	UC [1]	Patients	Percent	Surgeons	Month first used
**Exeter X3 Rimfit**		**13,821**	**38.5%**	**781**	**Jun 2010**
**Trinity**	✓	**6,133**	**17.1%**	**197**	**Nov 2009**
**Pinnacle Gription**	✓	**2,817**	**7.9%**	**375**	**Dec 2009**
**Delta TT**	✓	**2,368**	**6.6%**	**145**	**Jun 2009**
**DeltaMotion**	✓	**1,823**	**5.1%**	**152**	**Feb 2009**
Versafit CC Trio	✓	1,442	4.0%	47	Mar 2011
RM Pressfit Vitamys	✓	731	2.0%	33	Aug 2011
G7 Cementless Acetabular Component	✓	621	1.7%	36	Aug 2014
AEON Cemented Acetabular Cup		568	1.6%	43	Sep 2011
Exceed ABT Cemented		556	1.5%	57	Jun 2011
Plasmafit Cementless Cup	✓	546	1.5%	50	Nov 2012
Duracel		465	1.3%	45	Mar 2013
Allofit IT	✓	367	1.0%	19	Jan 2010
ADES Cemented		342	1.0%	72	Feb 2014
XLFit Acetabular Cup	✓	293	0.8%	56	Apr 2015
Regenerex Ringloc+	✓	220	0.6%	55	Feb 2009
April - Polyethylene	✓	214	0.6%	33	Jan 2012
ADES	✓	205	0.6%	39	May 2014
Delta PF	✓	198	0.6%	9	Mar 2011
MIHR Cup	✓	197	0.5%	12	Mar 2008
RM Pressfit	✓	184	0.5%	24	May 2008
Tribofit	✓	174	0.5%	9	Jul 2010
seleXys TH+	✓	174	0.5%	13	Nov 2008
OptiCup CEP		147	0.4%	18	Nov 2014
Delta One TT	✓	129	0.4%	61	Jun 2010
Gyros	✓	129	0.4%	28	Jan 2010
Restoration ADM Cup	✓	127	0.4%	31	May 2011
EcoFit Cementless Cup	✓	102	0.3%	5	Feb 2013
Novation	✓	93	0.3%	10	Nov 2009
Allofit-S IT	✓	91	0.3%	21	Aug 2010
M2A Magnum	✓	79	0.2%	30	Feb 2008
Freedom		75	0.2%	17	May 2008
Captiv DM	✓	68	0.2%	8	Aug 2011
Trident Constrained Cup		65	0.2%	30	Jan 2008

seleXys DS Cementless	✓	56	0.2%	16	Mar 2014
MMC Resurfacing	✓	36	0.1%	10	Aug 2009
ASR 300 Cup	✓	36	0.1%	1	Jan 2009
MPACT	✓	25	0.1%	8	Dec 2011
Restoration Gap2		23	0.1%	14	Mar 2008
Fixa Ti-Por	✓	20	0.1%	4	Apr 2014
seleXys DS Cemented		20	0.1%	12	Feb 2014
Fixa Duplex	✓	17	0.0%	1	Mar 2016
Cormet Prime	✓	12	0.0%	5	Jan 2010
Delta Revision TT	✓	9	0.0%	6	Nov 2010
Equateur	✓	9	0.0%	5	Jul 2008
U-Motion II	✓	8	0.0%	4	Apr 2016
A Class		7	0.0%	3	Feb 2009
Zimmer Cemented Cup		6	0.0%	4	May 2013
Regenerex Revision	✓	4	0.0%	3	Jan 2009
Capitole C		3	0.0%	3	Jan 2013
Sirius Cementless Cup	✓	3	0.0%	2	Aug 2011
Horizon	✓	3	0.0%	2	Jul 2008
2M Dual Mobility	✓	3	0.0%	2	Nov 2012
Par-5	✓	3	0.0%	3	Jan 2008
Solution Cemented Cup		2	0.0%	1	Dec 2015
J-Loc	✓	2	0.0%	2	Mar 2013
XPE Cup		2	0.0%	1	Jun 2016
Evidence		2	0.0%	1	Oct 2014
FIXA Duplex Cemented		1	0.0%	1	Jan 2017
Endurance Cemented Cup		1	0.0%	1	Oct 2016
Mitre Cup		1	0.0%	1	Nov 2013
Capitole T	✓	1	0.0%	1	Nov 2014
Polymax	✓	1	0.0%	1	Oct 2016
Ringloc	✓	1	0.0%	1	Jan 2011
Charnley KS		1	0.0%	1	Jul 2011
Arden		1	0.0%	1	Feb 2008
Versacem		1	0.0%	1	Oct 2009
Versafit DM	✓	1	0.0%	1	May 2008
Total		35,885			

1 – Uncemented fixation, Rows in **bold** = five most commonly used new cups

Table S2 Uptake of new stems first used between January 1st 2008 and 26th February 2017

Stem brand	UC [1]	Patients	Percent	Surgeons	Month first used
**Trilock BPS**	✓	**2,232**	**12.3%**	**121**	**Dec 2009**
**Accolade II**	✓	**1,997**	**11.0%**	**165**	**Jan 2012**
**Polarstem Cementless**	✓	**1,969**	**10.8%**	**93**	**Dec 2008**
**Taperloc Complete Cementless Stem**	✓	**1,658**	**9.1%**	**104**	**Jan 2011**
**miniHip**	✓	**1,193**	**6.6%**	**79**	**Mar 2009**
Metafix Stem	✓	1,171	6.4%	93	Feb 2008
AMIStem-H	✓	1,003	5.5%	32	Aug 2009
Exeter No.1 125mm stem Line Extension		836	4.6%	211	Aug 2014
TriFit TS hip stem	✓	684	3.8%	44	Sep 2012
Aeon Cemented Stem		673	3.7%	50	Sep 2011
SPS Evolution	✓	654	3.6%	48	Jan 2012
C-Stem AMT Line Extension		428	2.4%	127	Jul 2013
H-Max S Monoblock Stem	✓	401	2.2%	34	May 2010
H-Max M Modular Stem	✓	316	1.7%	20	Mar 2010
Finsbury Type C	✓	302	1.7%	39	Aug 2008
EcoFit Cementless Stem	✓	240	1.3%	11	Sep 2010
Silent	✓	199	1.1%	17	Feb 2008
Metha Monoblock Stem	✓	195	1.1%	25	Aug 2011
Corail Cemented		170	0.9%	32	Apr 2009
OptiStem		165	0.9%	22	Nov 2014
Trilliance		156	0.9%	10	Jul 2011
Sirius stem		138	0.8%	10	Apr 2014
Profemur L Classic	✓	132	0.7%	17	Mar 2014
Profemur TL	✓	120	0.7%	23	Jan 2008
AMIStem-C		110	0.6%	3	Jul 2012
Master SL	✓	102	0.6%	8	Jul 2013
Corail Revision Stem	✓	92	0.5%	69	Jul 2010
Novation Element Stem	✓	90	0.5%	9	Nov 2009
CBC Evolution	✓	83	0.5%	8	Jan 2013
Nanos	✓	78	0.4%	5	Dec 2011
Amoda	✓	67	0.4%	1	Apr 2010
Harmony Modular	✓	65	0.4%	6	Mar 2010
ABG II Cementless Stem	✓	52	0.3%	9	Apr 2009
SL	✓	51	0.3%	5	Sep 2009
XActa		47	0.3%	5	Jan 2014
Avenir Muller Cementless	✓	33	0.2%	6	Jun 2016
Harmony Cemented		25	0.1%	8	Feb 2014

miniMax	✓	24	0.1%	2	Apr 2011
SMS	✓	22	0.1%	2	Jul 2015
FTS	✓	20	0.1%	5	Feb 2009
Profemur TL Classic	✓	19	0.1%	5	Jan 2016
SMF	✓	17	0.1%	1	Oct 2011
Profemur Preserve	✓	12	0.1%	5	Feb 2012
AMIStem HP	✓	12	0.1%	1	Dec 2015
METS Cemented		12	0.1%	10	Dec 2012
GMRS		11	0.1%	9	Aug 2012
Harmony Cementless	✓	10	0.1%	4	Apr 2011
UCP Stem		10	0.1%	5	Apr 2016
Echelon Cemented Stem		8	0.0%	6	Mar 2008
Exception Cementless	✓	6	0.0%	3	Feb 2010
METS Cementless	✓	5	0.0%	5	Feb 2013
Novation Stem	✓	5	0.0%	2	Mar 2014
G2 Cementless Stem	✓	5	0.0%	5	Dec 2013
Securus	✓	4	0.0%	4	Dec 2009
Profemur Gladiator	✓	4	0.0%	3	Mar 2010
Arcad Cementless	✓	4	0.0%	4	Sep 2010
Euros Cementless	✓	3	0.0%	2	Aug 2011
Atlantis	✓	3	0.0%	3	Dec 2011
Quadra-C		3	0.0%	2	Oct 2009
Restoration Cemented Stem		1	0.0%	1	Feb 2014
Wagner Revision Stem	✓	1	0.0%	1	Apr 2016
Initiale Cemented Stem		1	0.0%	1	Jul 2008
Integrale	✓	1	0.0%	1	Jun 2009
optimys	✓	1	0.0%	1	Feb 2017
Prodigy	✓	1	0.0%	1	Jul 2010
CDH Stem	✓	1	0.0%	1	Nov 2012
Friendly		1	0.0%	1	Jul 2012
Regulus Cemented Stem		1	0.0%	1	Oct 2016
Arcad Cemented		1	0.0%	1	Feb 2009
Endurance Cemented Stem		1	0.0%	1	Sep 2013
Furlong HAC Hemiarthroplasty	✓	1	0.0%	1	Oct 2010
C2 Stem	✓	1	0.0%	1	Feb 2015
**Total**		**18,159**			

1 – Uncemented fixation, Rows in **bold** = five most commonly used new stems

Table S3 Number of different post-2008 shells/cups used by surgeons

Number of new cups used	Number of surgeons	Percent	Cumulative percent
1	1,113	65.5%	65.5%
2	351	20.7%	86.2%
3	138	8.1%	94.3%
4	61	3.6%	97.9%
5	18	1.1%	98.9%
6	8	0.5%	99.4%
7	9	0.5%	99.9%
10	1	0.1%	100.0%
**Total**	1,699		

Table S4 Number of different post-2008 stems used by surgeons

Number of new stems used	Number of surgeons	Percent	Cumulative percent
1	771	69.4%	69.4%
2	210	18.9%	88.3%
3	77	6.9%	95.2%
4	33	3.0%	98.2%
5	9	0.8%	99.0%
6	8	0.7%	99.7%
7	1	0.1%	99.8%
8	2	0.2%	100.0%
**Total**	1,111		

Table S5 The number of unique new stem-cup combinations used simultaneously by surgeons

**Stem-cup combinations**	**Number of surgeons**	**Percent**	**Cumulative percent**
1	311	71.8%	71.8%
2	78	18.0%	89.8%
3	22	5.1%	94.9%
4	10	2.3%	97.2%
5	5	1.2%	98.4%
6	2	0.5%	98.9%
7	1	0.2%	99.1%
8	2	0.5%	99.5%
9	2	0.5%	100.0%
**Total**	433	100%	

Table S6 A comparison of people with complete data and those missing some data

	Incomplete (n=186,438)	Complete (n=431,995)	P*
**Age**			
<55 years old	19,561 (10.5%)	46,573 (10.8%)	<0.001
55 to 80	136,370 (73.1%)	321,451 (74.4%)	
≥ 80 years old	30,507 (16.4%)	63,931 (14.8%)	
**Gender**			
Male	71,676 (38.4%)	171,375 (39.7%)	<0.001
Female	114,762 (61.6%)	260,580 (60.3%)	
**BMI**			
Underweight and normal	31 (23.8%)	98,217 (22.7%)	0.097
Overweight	43 (33.1%)	170,987 (39.6%)	
Class I Obese	33 (25.4%)	109,061 (25.2%)	
Class II Obese	14 (10.8%)	40,271 (9.3%)	
Class III Obese	9 (6.9%)	13,419 (3.1%)	
**ASA grade**			
I	24,893 (13.4%)	62,683 (14.5%)	<0.001
II	130,223 (69.8%)	302,082 (69.9%)	
III	30,180 (16.2%)	65,386 (15.1%)	
IV + V	1,142 (0.6%)	1,804 (0.4%)	
**Source of funding**			
NHS	165,394 (89.4%)	375,481 (86.9%)	<0.001
Private	19,530 (10.6%)	5,6474 (13.1%)	

*- P-values from chi square tests

Table S7 Results from unadjusted logistic regression models showing the association between surgeon-level factors and use of new versus old stems and cups

	**Stems**			**Cups**		
**Exposure**	**OR[1]**	**(95% CI)**	**p**	**OR[1]**	**(95% CI)**	**p**
**Proportion of THRs performed on patients <55 years old**						
<10% (ref.)	1	-	-	1	-	-
≥10%	2.79	2.49 – 3.13	<0.001	2.38	2.16 – 2.62	<0.001
**Number of THRs performed in calendar year[2]** (per 10 additional cases)	1.19	1.16 – 1.22	<0.001	1.20	1.18 – 1.23	<0.001
**Proportion of THRs funded privately**						
100% NHS funded (ref.)	1	-	-	1	-	-
Some or all funded privately	2.54	2.21 – 2.91	<0.001	2.13	1.90 – 2.37	<0.001
**Number of stem-cup combinations used in calendar year**						
≤3 (ref.)	1	-	-	1	-	-
4-6	5.86	5.16 – 6.66	<0.001	4.74	4.27 – 5.26	<0.001
7-10	16.8	14.1 – 20.0	<0.001	11.0	9.39 – 12.9	<0.001
>10	40.5	27.2 – 60.1	<0.001	21.2	17.1 – 34.2	<0.001
**Proportion of THRs performed on patients with ASA grade III-V**						
<25% (ref.)	1	-	-	1	-	-
≥25%	0.75	0.66 – 0.85	<0.001	0.85	0.77 – 0.95	0.003

1 – odds ratios, 95% confidence intervals and p-values are from unadjusted logistic regression models

Table S8 Results from unadjusted mixed-effects regression models (patients nested within surgeons) of age, gender, categorised BMI, ASA grade, and source of funding on stem and cup age

	**Stems**			**Cups**		
	**OR[1]**	**(95% CI)**	**p**	**OR[1]**	**(95% CI)**	**p**
**Age** (years)						
<55 years old	1.95	1.85 – 2.05	<0.001	1.31	1.25 – 1.36	<0.001
55 to 80 (ref.)	1	-	-	1	-	-
≥ 80 years old	0.56	0.52 – 0.60	<0.001	0.93	0.89 – 0.97	<0.001
**Gender**						
Male (ref.)	1	-	-	1	-	-
Female	0.83	0.80 – 0.86	<0.001	1.05	1.02 – 1.08	0.001
**BMI**						
Underweight and normal (ref.)	1	-	-	1	-	-
Overweight	1.06	1.01 – 1.12	0.017	0.94	0.91 – 0.98	0.001
Class I Obese	1.12	1.06 – 1.18	<0.001	0.93	0.90 – 0.97	0.001
Class II Obese	1.18	1.09 – 1.27	<0.001	0.96	0.90 – 1.01	0.123
Class III Obese	1.04	0.93 – 1.17	0.513	0.97	0.89 – 1.05	0.456
**ASA grade**						
I (ref.)	1	-	-	1	-	-
II	0.69	0.65 – 0.72	<0.001	0.92	0.88 – 0.96	<0.001
III	0.51	0.48 – 0.55	<0.001	0.90	0.86 – 0.95	<0.001
IV + V	0.47	0.33 – 0.65	<0.001	0.91	0.74 – 1.13	0.390
**Source of funding**						
NHS	1	-	-	1	-	-
Private	1.01	0.95 – 1.07	0.777	1.08	1.03 – 1.13	0.001

1 – odds ratios, 95% confidence intervals and p-values are from unadjusted mixed-effects logistic regression models

Table S9 Sensitivity analysis 1: Results from multivariable logistic regression models showing the association between surgeon-level factors and use of new versus old stems and cups, excluding surgeon calendar-years with <10 THRs

	Stems			Cups		
**Exposure**	**OR[1]**	**(95% CI)**	**p**	**OR[1]**	**(95% CI)**	**p**
**Proportion of THRs performed on patients <55 years old**						
<10% (ref.)	1	-	-	1	-	-
≥10%	1.30	1.13 – 1.49	<0.001	1.36	1.21 – 1.54	<0.001
**Number of THRs performed in calendar year[2]** (per 10 additional cases)	1.01	0.99 – 1.04	0.359	1.04	1.01 – 1.06	0.001
**Proportion of THRs funded privately**						
100% NHS funded (ref.)	1	-	-	1	-	-
Some or all funded privately	1.24	1.05 – 1.47	0.012	1.04	0.91 – 1.19	0.546
**Number of stem-cup combinations used in calendar year**						
≤3 (ref.)	1	-	-	1	-	-
4-6	4.13	3.44 – 4.96	<0.001	3.22	2.82 – 3.69	<0.001
7-10	10.8	8.62 – 13.6	<0.001	6.23	5.15 – 7.53	<0.001
>10	24.3	15.8 – 37.4	<0.001	12.1	8.37 – 17.5	<0.001
**Proportion of THRs performed on patients with ASA grade III-V**						
<25% (ref.)	1	-	-	1	-	-
≥25%	1.11	0.94 – 1.31	0.214	1.21	1.05 – 1.38	0.007

1 – odds ratios, 95% confidence intervals and p-values are from logistic regression models adjusted for all exposure variables

Table S10Sensitivity analysis 2a: Results from multivariable logistic regression models showing the association between surgeon-level factors (consultant in-charge) and use of new versus old stems and cups

	Stems			Cups		
**Exposure**	**OR[1]**	**(95% CI)**	**p**	**OR[1]**	**(95% CI)**	**p**
**Proportion of THRs performed on patients <55 years old**						
<10% (ref.)	1	-	-	1	-	-
≥10%	1.52	1.33 – 1.74	<0.001	1.46	1.30 – 1.64	<0.001
**Number of THRs performed in calendar year[2]** (per 10 additional cases)	1.02	1.00 – 1.04	0.039	1.05	1.04 – 1.07	<0.001
**Proportion of THRs funded privately**						
100% NHS funded (ref.)	1	-	-	1	-	-
Some or all funded privately	1.27	1.08 – 1.50	0.004	1.15	1.01 – 1.31	0.042
**Number of stem-cup combinations used in calendar year**						
≤3 (ref.)	1	-	-	1	-	-
4-6	4.72	3.98 – 5.60	<0.001	3.47	3.04 – 3.97	<0.001
7-10	11.2	8.87 – 14.1	<0.001	6.16	5.06 – 7.49	<0.001
>10	26.7	17.9 – 39.9	<0.001	11.3	7.91 – 16.1	<0.001
**Proportion of THRs performed on patients with ASA grade III-V**						
<25% (ref.)	1	-	-	1	-	-
≥25%	1.15	0.98 – 1.35	0.077	1.13	0.99 – 1.29	0.064

1 – odds ratios, 95% confidence intervals and p-values are from logistic regression models adjusted for all exposure variables

Table S11 Sensitivity analysis 2b: Results from multivariable mixed-effects regression models (patients nested within 'consultant in-charge') of age, gender, categorised BMI, ASA grade, and source of funding on stem and cup age

	**Stems**			**Cups**		
	**OR[1]**	**(95% CI)**	**p**	**OR[1]**	**(95% CI)**	**p**
**Age** (years)						
<55 years old	1.89	1.79 – 1.99	<0.001	1.32	1.26 – 1.37	<0.001
55 to 80 (ref.)	1	-	-	1	-	-
≥ 80 years old	0.59	0.55 – 0.63	<0.001	0.92	0.88 – 0.96	<0.001
**Gender**						
Male (ref.)	1	-	-	1	-	-
Female	0.87	0.84 – 0.91	<0.001	1.05	1.02 – 1.09	<0.001
**BMI**						
Underweight and normal (ref.)	1	-	-	1	-	-
Overweight	1.01	0.96 – 1.07	0.663	0.95	0.91 – 0.98	0.005
Class I Obese	1.05	0.99 – 1.11	0.123	0.93	0.89 – 0.97	<0.001
Class II Obese	1.09	1.01 – 1.17	0.031	0.94	0.89 – 1.00	0.039
Class III Obese	0.98	0.87 – 1.10	0.737	0.95	0.87 – 1.04	0.264
**ASA grade**						
I (ref.)	1	-	-	1	-	-
II	0.80	0.76 – 0.84	<0.001	0.97	0.93 – 1.01	0.168
III	0.64	0.60 – 0.69	<0.001	0.97	0.92 – 1.03	0.338
IV + V	0.62	0.44 – 0.87	0.006	1.00	0.81 – 1.23	0.981
**Source of funding**						
NHS	1	-	-	1	-	-
Private	1.14	1.07 – 1.21	<0.001	1.13	1.08 – 1.18	<0.001

1 – odds ratios, 95% confidence intervals and p-values are from mixed-effects logistic regression models adjusted for all exposure variables

Table S11 Sensitivity analysis 3: A comparison of results from multivariable mixed-effects regression models (patients nested within 'lead surgeon', excluding BMI) using a) patients with complete data for all exposures and BMI and, b) patients with complete data for all exposures excluding BMI

	**Stems**						**Cups**					
	**a) Complete cases only[1] (n = 431,955)**			**b) All cases[2] (n = 616,879)**			**a) Complete cases only[1] (n = 431,955)**			**b) All cases[2] (n = 616,879)**		
	**OR[3]**	**(95% CI)**	**p**	**OR[3]**	**(95% CI)**	**p**	**OR[3]**	**(95% CI)**	**p**	**OR[3]**	**(95% CI)**	**p**
**Age** (years)												
<55 years old	1.83	1.74 – 1.93	<0.001	1.81	1.73 – 1.90	<0.001	1.31	1.25 – 1.37	<0.001	1.37	1.32 – 1.42	<0.001
55 to 80 (ref.)	1	-	-	1	-	-	1	-	-	1	-	-
≥ 80 years old	0.59	0.55 – 0.63	<0.001	0.61	0.61 – 0.57	<0.001	0.92	0.88 – 0.96	<0.001	0.95	0.92 – 0.99	0.008
**Gender**												
Male (ref.)	1	-	-	1	-	-	1	-	-	1	-	-
Female	0.86	0.83 – 0.90	<0.001	0.84	0.82 – 0.87	<0.001	1.07	1.04 – 1.10	<0.001	1.04	1.02 – 1.07	0.001
**ASA grade**												
I (ref.)	1	-	-	1	-	-	1	-	-	1	-	-
II	0.82	0.78 – 0.87	<0.001	0.82	0.78 – 0.86	<0.001	0.98	0.94 – 1.02	0.264	0.97	0.94 – 1.01	0.100
III	0.67	0.62 – 0.72	<0.001	0.66	0.62 – 0.70	<0.001	0.99	0.94 – 1.04	0.641	1.01	0.97 – 1.06	0.591
IV + V	0.65	0.46 – 0.91	0.011	0.67	0.52 – 0.86	0.002	1.01	0.82 – 1.25	0.927	1.12	0.95 – 1.32	0.175
**Source of funding**												
NHS (ref.)	1	-	-	1	-	-	1	-	-	1	-	-
Private	1.01	0.95 – 1.08	0.688	1.02	0.97 – 1.08	0.403	1.09	1.04 – 1.14	<0.001	1.07	1.03 – 1.11	0.001

1 – The study sample for 'Complete cases only' was defined as those cases with complete data for all exposure variables (age, gender, ASA grade and source of funding) and BMI

2 - The study sample for 'All cases' was defined as those cases with complete data for all exposure variables (age, gender, ASA grade and source of funding)

3 – odds ratios, 95% confidence intervals and p-values are from mixed-effects logistic regression models adjusted for all exposure variables

Figure S1 The cumulative introduction of new brands of cup and stem components for THRs, between January 1st 2008 and 26th February 2017

Figure S2 STROBE Flow diagram
