# Supplementary figures and images for: Understanding the uptake of new hip replacement implants in the UK: a cohort study using data from the National Joint Registry for England and Wales
Source: BMJ Open. 2019 Nov 25;9(11):e029572. doi: 10.1136/bmjopen-2019-029572 (PMC6886938; doi:10.1136/bmjopen-2019-029572)

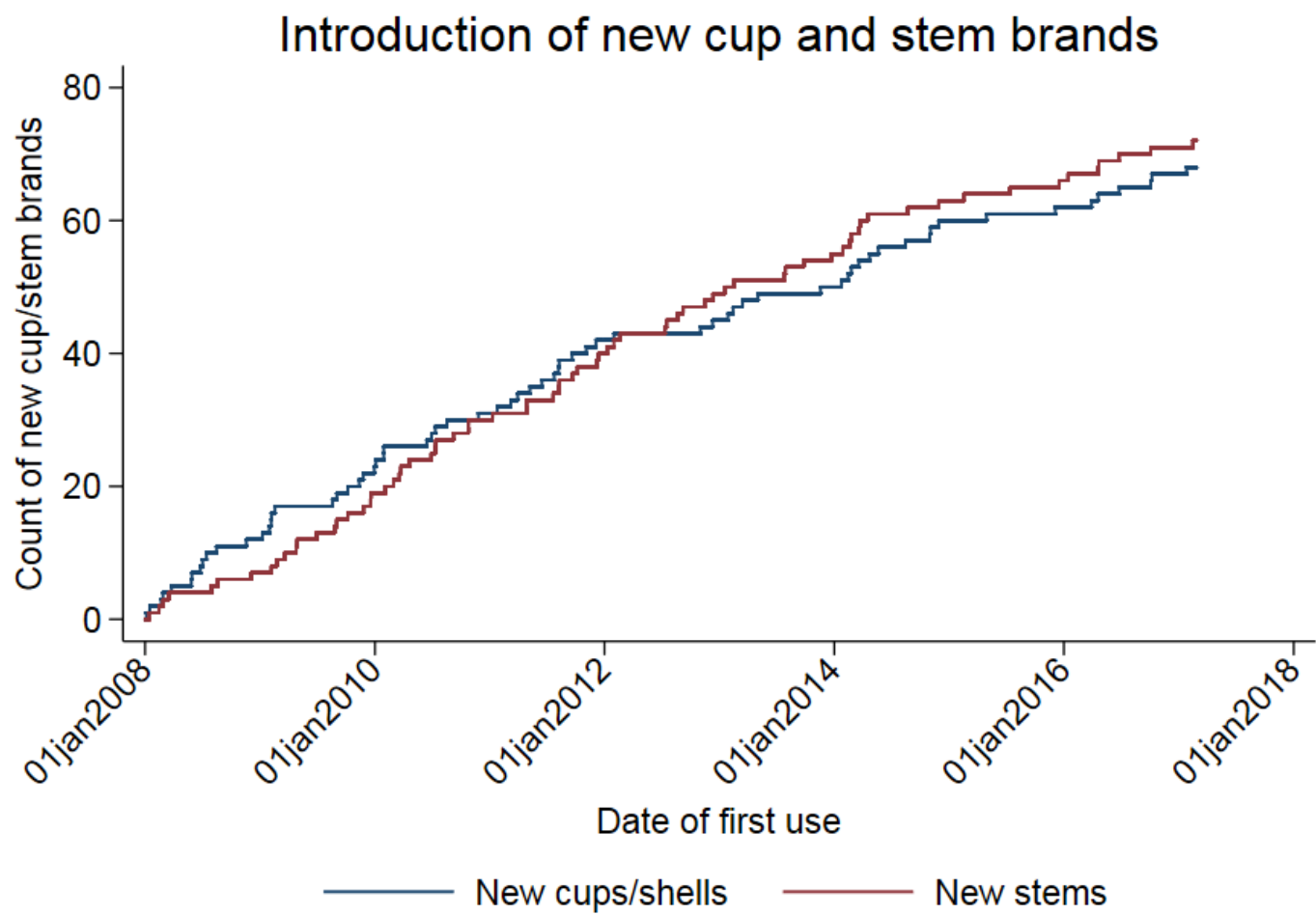

Supplement: Supplementary data [file bmjopen-2019-029572supp002.pdf]

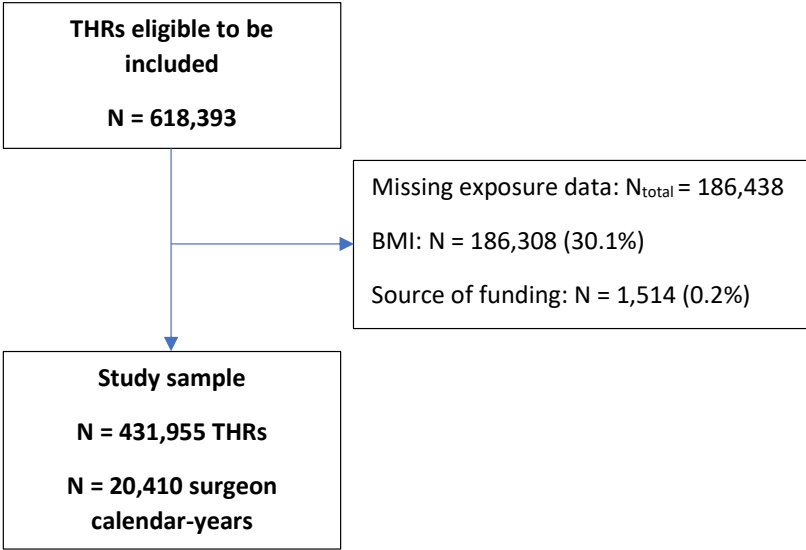

Supplement: Supplementary data [file bmjopen-2019-029572supp003.pdf]
