# Supplementary material for: Understanding the uptake of new hip replacement implants in the UK: a cohort study using data from the National Joint Registry for England and Wales
Source: BMJ Open. 2019 Nov 25;9(11):e029572. doi: 10.1136/bmjopen-2019-029572 (PMC6886938; doi:10.1136/bmjopen-2019-029572)
Supplement: Author's manuscript [file bmjopen-2019-029572.draft_revisions.pdf]

BMJ Open

BMJ Open is committed to open peer review. As part of this commitment we make the peer review history of every article we publish publicly available.

When an article is published we post the peer reviewers' comments and the authors' responses online. We also post the versions of the paper that were used during peer review. These are the versions that the peer review comments apply to.

The versions of the paper that follow are the versions that were submitted during the peer review process. They are not the versions of record or the final published versions. They should not be cited or distributed as the published version of this manuscript.

BMJ Open is an open access journal and the full, final, typeset and author-corrected version of record of the manuscript is available on our site with no access controls, subscription charges or pay-per-view fees (http://bmjopen.bmj.com).

If you have any questions on BMJ Open's open peer review process please email info.bmjopen@bmj.com

BMJ Open

Understanding the uptake of new hip replacement implants in the UK: Analysis of data from the National Joint Registry for England and Wales

Journal:	*BMJ Open*
Manuscript ID	bmjopen-2019-029572
Article Type:	Research
Date Submitted by the Author:	31-Jan-2019
Complete List of Authors:	Penfold, Chris; University of Bristol, Musculoskeletal Research Unit, Translational Health Sciences, Bristol Medical School; University Hospitals Bristol NHS Foundation Trust and University of Bristol, National Institute for Health Research Bristol Biomedical Research Centre<br>Blom, AW; University of Bristol, Musculoskeletal Research Unit, Translational Health Sciences, Bristol Medical School; University Hospitals Bristol NHS Foundation Trust and University of Bristol, National Institute for Health Research Bristol Biomedical Research Centre<br>Sayers, Adrian; University of Bristol, Muscloskeletal Research Unit, Translational Health Sciences, Bristol Medical School; University of Bristol, Population Health Sciences, Bristol Medical School<br>Wilkinson, J. Mark; Northern General Hospital, Metabolic Bone Unit, Sorby Wing; University of Sheffield, 5. Department of Oncology and Metabolism and The Mellanby Centre for Bone Research<br>Hunt, Linda; University of Bristol, Musculoskeletal Research Unit, Translational Health Sciences, Bristol Medical School<br>Judge, Andrew; University of Bristol, Musculoskeletal Research Unit, Translational Health Sciences, Bristol Medical School; University Hospitals Bristol NHS Foundation Trust and University of Bristol, National Institute for Health Research Bristol Biomedical Research Centre<br>Whitehouse, Michael; University of Bristol, Musculoskeletal Research Unit, Translational Health Sciences, Bristol Medical School; University Hospitals Bristol NHS Foundation Trust and University of Bristol, National Institute for Health Research Bristol Biomedical Research Centre
Keywords:	Orthopaedic & trauma surgery < SURGERY, Joint replacement, Implant, Patient, Surgeon, National Joint Registry

SCHOLARONE™
Manuscripts

Understanding the uptake of new hip replacement implants in the UK: Analysis of data from the National Joint Registry for England and Wales

Chris M Penfold[1+2*]

Ashley W Blom[1+2]

Adrian Sayers[1+3]

J Mark Wilkinson[4+5]

Linda P Hunt[1]

Andrew Judge[1+2]

Michael R Whitehouse[1+2]

Affiliations:

1. Musculoskeletal Research Unit, Translational Health Sciences, Bristol Medical School, 1st Floor Learning & Research Building, Southmead Hospital, Bristol, BS10 5NB, UK
2. National Institute for Health Research Bristol Biomedical Research Centre, University Hospitals Bristol NHS Foundation Trust and University of Bristol.
3. Population Health Sciences, Bristol Medical School, University of Bristol, Bristol
4. Metabolic Bone Unit, Sorby Wing, Northern General Hospital, Sheffield, S5 7AU, UK
5. Department of Oncology and Metabolism and The Mellanby Centre for Bone Research, University of Sheffield

For peer review only

For peer review only - http://bmjopen.bmj.com/site/about/guidelines.xhtml

* - denotes corresponding author

Corresponding author contact details:

Email: chris.penfold@bristol.ac.uk

Address: Musculoskeletal Research Unit, University of Bristol, School of Clinical Sciences, Learning and Research Building, Southmead Hospital, Bristol, BS10 5NB

Telephone: 0117 41 47872

Keywords

Orthopaedics, joint replacement, implant, patient, surgeon, national joint registry

Data sharing statement

Access to the data analysed in this study required permission from the National Joint Registry for England, Wales and Northern Ireland Research Sub-committee. http://www.njrcentre.org.uk/njrcentre/Research/Researchrequests/tabid/305/Default.aspx contains information on research data access request to the National Joint Registry.

Acknowledgements

We thank the patients and staff of all the hospitals who have contributed data to the National Joint Registry. We are grateful to the Healthcare Quality Improvement Partnership, the National Joint Registry Steering Committee, and staff at the National Joint Registry for facilitating this work.

Disclaimer

The views expressed represent those of the authors and do not necessarily reflect those of the National Joint Registry Steering Committee or Healthcare Quality Improvement Partnership, who do not vouch for how the information is presented. The views expressed in this article are those of the authors and not necessarily those of the NHS, the NIHR, or the Department of Health and Social Care.

For peer review only - http://bmjopen.bmj.com/site/about/guidelines.xhtml

Ethics approval

Patient consent was obtained for data collection by the National Joint Registry. According to the specifications of the NHS Health Research Authority, separate informed consent and ethical approval were not required for the present study.

Author Contributions

CP, AB, AJ and MW designed the study. CP, AB, AS, JMW, LH, AJ and MW reviewed the published work. CP conducted the statistical analysis and wrote the report. CP had full access to all the data and AB is the guarantor.

Conflicts of Interest

Competing interests: All authors have completed the ICMJE uniform disclosure form at www.icmje.org/coi_disclosure.pdf and declare: no support from any organisation for the submitted work. AB and MW are involved in a separate grant to the University of Bristol funded by Stryker. All other authors declare no financial relationships with any organisations that might have an interest in the submitted work in the previous three years. All authors declare no other relationships or activities that could appear to have influenced the submitted work.

Funding

This study was funded by the NIHR Biomedical Research Centre at University Hospitals Bristol NHS Foundation Trust and the University of Bristol. The views expressed in this publication are those of the authors and not necessarily those of the NHS, the National Institute for Health Research or the Department of Health and Social Care. Adrian Sayers was supported by a MRC fellowship MR/L01226X/1.

Word count

3213 words

For peer review only - http://bmjopen.bmj.com/site/about/guidelines.xhtml

For peer review only

Abstract

Objectives

Primary: to describe the uptake of new implant components (femoral stem or acetabular cup/shell) for total hip replacements (THRs) in the National Joint Registry for England and Wales (NJR). Secondary: to compare the characteristics of: a) surgeons and b) patients who used/received new rather than established components.

Design

A cohort of 618,393 primary THRs performed for osteoarthritis (±other indications) by 4,979 surgeons between 2008-2017 in England and Wales from the NJR. We described the uptake of new (first recorded in NJR >2008) stems/cups, and variation in uptake by operating surgeons (primary objectives). We explored surgeon-level and patient-level factors associated with use/receipt of new components with logistic regression models (secondary objectives).

Outcomes

Primary outcomes: total number of new cups/stems, and proportion of operations using new versus established components. Secondary outcomes: odds of: a) a surgeon using a new cup/stem in a calendar-year, b) a patient receiving a new rather than established cup/stem.

Results

Sixty-eight new cups and 72 new stems were used in 72,349 primary THRs (11.7%) by 2,423 surgeons (48.7%) 2008-2017. Surgeons used a median of one new stem and cup (IQR=1-2 both, max=11 cups, max=9 stems). Surgeons performed a median of 22 THRs (IQR 5-124, range 1-3,938), a median of 5.0% (IQR 1.3-16.1%) and 9.4% (IQR 2.8-26.7%) used new stems and cups respectively. Patients aged <55 years old versus those 55-80 had higher odds of receiving a new rather than established stem (OR=2.13, 95%CI 2.04-2.23) and cup (OR=1.40, 95%CI 1.34-1.45). Women had lower odds of receiving a new stem (OR=0.81, 95%CI 0.78-0.84), higher odds of receiving a new cup (OR=1.11, 95%CI 1.08-1.14).

Conclusions

Large numbers of new THR components have been introduced in the NJR since 2008. Half of surgeons have tried new components, with wide variation in how many types and how often they have been used.

For peer review only - http://bmjopen.bmj.com/site/about/guidelines.xhtml

For peer review only

Article Summary

Strengths and limitations of this study

- This study provides a nationally representative description of the uptake of new implant components for total hip replacements in England and Wales.
- This is the first study to describe the variation in uptake of new components by surgeons, and surgeon characteristics which may be associated with the use of new components.
- Although implant component brand names were checked by the authors, some components may have been reclassified or we may still have misclassified some components as either new or established, but the introduction of unique device identifiers should remove this problem in future.
- The surgeon assigned as lead operating surgeon in the NJR may not be correct, although consistency between our sensitivity and primary analyses indicate that this is unlikely to have substantially affected our findings.
- Hospital-level or regional variation in suppliers may be important factors affecting implant uptake, but these were beyond the scope of this study.

For peer review only

For peer review only - http://bmjopen.bmj.com/site/about/guidelines.xhtml

Background

Total hip replacements (THRs) are mainly performed to treat pain and functional limitation due to osteoarthritis (OA).[1] It is a highly successful surgical procedure with typical 10-year revision rates <5%,[2] the current NICE benchmark.[3] However, younger patients are more likely to require revision surgery; the lifetime revision risk for men having a THR in their 50s is ~35% compared with 5% in their 70s.[4] Such patients may benefit the most from developments in THR that lead to reduced revision rates or improved outcomes.

Some new implant designs intended to benefit these more active and/or younger patients have been high-profile failures, for example metal-on-metal THRs [5] including the Articular Surface Replacement (ASR) prostheses in particular.[6] Many new implants, the ASR included,[7] were introduced with minimal supporting evidence of their effectiveness [8] and may offer at best no improvement over pre-existing components.[9] An influential agenda for surgery research (IDEAL) was developed, providing a framework for future investigations into surgical innovations, which recommended the phased introduction of new medical devices.[10] The rapid uptake of metal-on-metal THRs was found to be inconsistent with this, it is not clear whether the introduction of newer implants since has followed the framework.

There is wide variation between and within regions of common surgical procedures.[11] The large number of different components used in primary THRs (127 femoral stems and 105 acetabular cups recorded in the National Joint Registry for England, Wales, Northern Ireland and the Isle of Man (NJR) in 2016) [12] may be an important source of variation. Many registries describe the volume of different implant components used annually but not the variation in uptake of new implants between surgeons or which patients receive them. More research is needed to understand and reduce avoidable variation in outcomes created by differences in surgical activity.

We aimed to:

1. Describe the uptake of new implants for THRs in the NJR
2. Describe how this uptake varies by surgeons
3. Compare surgeons who use new compared with established components
4. Compare patients who receive new compared with established components

For peer review only - http://bmjopen.bmj.com/site/about/guidelines.xhtml

Methods

Data Source

The NJR was established in 2003.[2] Data entry for Northern Ireland and the Isle of Man did not commence until 2013 and 2015, respectively therefore they are excluded from this analysis.

Study sample

We included the cohort of patients who received a primary THR for OA (± other indications) between 1st January 2008 and 26th February 2017. We used NJR data from 2003 onwards to calculate the date each implant component was first used and the total number of implantations. We excluded people who had not given consent for recording of personal details, those who received a resurfacing rather than stemmed THR, and where the brand of their acetabular or femoral components was uncertain.

Patient involvement

This study was designed and undertaken without patient involvement.

Definition of new and established implant components

We identified the implant component brand from component labels recorded in the NJR and categorised all femoral (stem) or acetabular (cup or shell) components with a first recorded use by any surgeon in the NJR on or after 1st January 2008 as 'new'. Components with a first recorded use before 2008 were categorised as 'established'.

Surgeon uptake of new implant components

All surgeons with operations recorded in the NJR are assigned an anonymised identifier and their role in the operation ("consultant in charge" or "operating") is recorded. We summarised each operating surgeon's activity across each calendar-year in which they performed ≥1 THR. We considered five potential surgeon-level factors which may be associated with use of a new component in a calendar-year: total volume of THRs performed in that year, proportion of those THRs performed on patients <55 years old (<10% and ≥10%), source of funding for THRs ('100% NHS funded' or 'some or all privately funded'), proportion of THRs performed on patients with an American Society of Anaesthesiologists (ASA) grade III-V (<25% and ≥25%), and the range of stem-cup combinations used in that calendar-year ('≤3', '4-6', '7-10' and '>10').

For peer review only - http://bmjopen.bmj.com/site/about/guidelines.xhtml

Patients receiving new implant components

We used date of surgery to order patients within implant components and within surgeons. We categorised patients according to whether the component they received was new or established. We considered five potential patient-level factors which may be associated with their receipt of new components: age at the time of THR (<55, 55-80, and 80+ years), gender, body mass index (BMI), ASA grade, and NHS or private funding.

Statistical analyses

We described the use of unique stems and cups in primary THRs performed since January 1st 2008, the cumulative use of new components in patients, and the count of surgeons who used new components. We also described the total number of all and new cups, stems, and combinations.

Surgeon-level factors

In analyses of surgeon-level and patient-level factors associated with use of or receipt of new implants we included only those people with complete exposure and outcome data for the surgeon-level and patient-level analysis models (i.e. complete case analysis). We assumed that data were missing at random but did not use multiple imputation to account for these missing data since there were no variables in the NJR dataset which were not already in our regression models and which may have carried information about the missing data (particularly BMI).

Our outcome was whether a surgeon used a new component at least once for a THR in a calendar-year (stems and cups analysed separately), unit of analysis was surgeon calendar-years and exposure variables were those surgeon-level factors defined previously. We used multivariable adjusted logistic regression models, accounting for the clustering of calendar-years within surgeons.

Patient-level factors

Our outcome was whether a patient received a new rather than established component (stems and cups analysed separately), unit of analysis was patients and exposure variables were those patient-level factors defined previously. Patient-level factors were included in multivariable adjusted mixed-effects logistic regression models, with patients nested within surgeons.

Sensitivity analyses

We conducted two sensitivity analyses. To determine whether the lack of variability in patients operated on by low volume surgeons affected our results we repeated our surgeon-level analysis excluding calendar-years for surgeons in which they performed <10 THRs. We also considered that the choice of

For peer review only - http://bmjopen.bmj.com/site/about/guidelines.xhtml

component was made by the consultant in-charge rather than the operating surgeon (the consultant in-charge was not the operating surgeon for ~16% of THRs). We repeated our surgeon-level analysis by consultant in-charge and repeated our patient-level analysis with patients clustered within consultant in-charge.

All analyses were performed using Stata v15 (StataCorp).

Results

Overall use of implant components

Between 1st January 2008 and 26th February 2017, 618,393 primary THRs were performed for OA in England and Wales and recorded in the NJR. The mean age of the patients was 68.5 years (SD=11.1 years), 60.7% were female, their ASA grades were I:14.2%, II:69.9%, III:15.5% and IV/V:0.5%. Twenty-three percent had a normal/underweight BMI, 39.6% were overweight and 37.6% obese. THRs were performed by 4,979 surgeons using 189 different stems, 187 cups and 2,026 stem-cup combinations. Surgeons used a median of three different stems (IQR=2-5, max=21), four cups (IQR=2-7, max=27) and five combinations (IQR=2-9, max=60), and performed a median of 22 THRs over the period (IQR 5-124, range 1-3,938).

Use of new implant components

During this period 68 new cups (47 uncemented, Table S1) and 72 new stems (51 uncemented, Table S2) were first used. The rate of introduction of new cups and stems remained stable (~16 new components/year, Figure S1). Twelve percent (n=72,349) of THRs performed used a new stem, cup, or combination. In 2016, 14.9% of THRs used a new cup (n=12,768/85,835) and nine percent a new stem (n=7,744, Figure 1). Forty eight percent (n=2,423) of surgeons who performed a THR in this period used at least one new implant component.

New cups were used in 9.0% (n=55,360) THRs performed by 41.5% (n=2,066) surgeons (Table S1), new stems in 4.7% (n=28,924) THRs by 26.4% (n=1,313) surgeons (Table S2) and new combinations in 1.9% (n=11,935) THRs by 12.5% (n=624) surgeons. Most new cups (n=31,448, 56.8%) and almost all new stems (n=25,684, 88.8%) were uncemented. The median number of new stems, cups and combinations used by surgeons was one (IQR=1-2, cups max=11, stems max=9 and combinations max=13; Tables S3-S5). The median THRs performed using new stems was three (IQR 1-12, max=769) and new cups was

For peer review only - http://bmjopen.bmj.com/site/about/guidelines.xhtml

four (IQR 1-17, max=1,211). The median proportion of a surgeon's THRs performed using new stems was 5.0% (IQR 1.3-16.1%), new cups 9.4% (IQR 2.8-26.7%) and new combinations 3.6% (IQR 0.9-13.3%).

The five most frequently implanted new stems were used in 18,528 THRs (63.1% of THRs using a new stem, Table S2). The five most frequently implanted new cups were used in 44,633 THRs (80.6% of THRs using a new cup, Table S1). Uptake of the two most popular new cups was rapid (5,000 uses of Exeter X3 Rimfit <1,000 days, 5,000 uses of Trinity ~1,500 days after first use, Figure 2) but was slower for new stems (5,000 uses of Polarstem Cementless ~2,800 days, Figure 2). Conversely, a third of the new stems and cups (n=24/72 new stems, n=24/69 new cups) have been used in ≤10 THRs.

Surgeon-level and patient-level factors associated with new implant components

Our complete case analysis included 431,955 out of a possible 618,393 THRs (69.8%) and 19,810 surgeon calendar-years (Figure S2). We were missing data for BMI (n=186,308, 30.1%) and source of funding (n=1,514, 0.2%).

Characteristics of surgeons using new implant components

Multivariable adjusted associations between surgeon-level factors and their use of new components in a calendar-year were consistent between stems and cups (Table 1, unadjusted Table S6). Surgeons who treated more younger patients had 51% higher odds of using a new stem (OR=1.51, 95%CI 1.34-1.69, p<0.001) and 45% higher odds of using a new cup (OR=1.45, 95%CI 1.32-1.59, p<0.001) in a calendar-year. Those who performed more THRs/year had 2% and 8% higher odds of using new stems (OR=1.02, 95%CI 1.00-1.05, p=0.03) and cups respectively (OR=1.08, 95%CI 1.05-1.10, p<0.001). Private funding was associated with 23% increased odds of using new stems (OR=1.23, 95%CI 1.06-1.42, p=0.006) and weakly associated with 11% increased odds of using new cups (OR=1.11, 95%CI 0.98-1.25, p=0.10). Use of more stem-cup combinations was strongly associated with increased use of new components (ORs for '>10' vs. '≤3' combinations: 23.3 and 13.9 for stems and cups respectively, p values <0.001). Proportion of patients with ASA grades III-IV was associated with 23% higher odds of using new cups (OR=1.23, 95%CI 1.12-1.35, P<0.001) but not with using new stems (OR=1.09, 95%CI 0.96-1.22, p=0.18).

Characteristics of patients receiving new implant components

A higher proportion of recipients of new compared with established implant components were aged <55 years old (10.2% established vs. 21.1% new stems; 10.4% established vs. 14.6% new cups; Table 2), although the main recipients of all components were aged 55-80 years. Fifteen percent of recipients of established stems (15.2%) were ≥80 years old compared with 7.6% of recipients of new stems, but there

For peer review only - http://bmjopen.bmj.com/site/about/guidelines.xhtml

was little difference in the proportion of older recipients of established (14.9%) and new (13.5%) cups. Across all components and component age, women were the main recipients of THRs. There was no difference in BMI between recipients of established and new stems or cups. A higher proportion of recipients of new components had ASA grade I (20.1% new vs. 14.2% established stems; 16.5% new vs. 14.3% established cups). A higher proportion of people with privately funded THRs had new components (stems: 18.4% new vs. 12.8% established; cups: 18.6% new vs. 12.5% established).

Multivariable adjusted mixed effects logistic regression models (Table 2, unadjusted Table S7) found that patients <55 years old, compared with those 55-80, had 113% and 40% higher odds of receiving a new rather than established stem (OR=2.13, 95%CI 2.04-2.23, p<0.001) and cup (OR=1.40, 95%CI 1.34-1.45, p<0.001). Women had 19% lower odds than men of receiving a new stem (OR=0.81, 95%CI 0.78-0.84, p<0.001), but 11% higher odds of receiving a new cup (OR=1.11, 95%CI 1.08-1.14, p<0.001). There was weak evidence that people with higher BMI had 14% higher odds of receiving a new stem (OR for underweight/normal vs. Class II Obese=1.14, 95%CI 1.07-1.22, p<0.001) but BMI was not associated with receiving a new cup (e.g. OR for underweight/normal vs. Class II Obese=0.97, 95%CI 0.93-1.02, p=0.29). Higher ASA grade was associated with 40% lower odds of receiving new stems (OR for ASA grades 'IV + V' versus 'I' = 0.60, 95%CI 0.45-0.80, p<0.001), but associated with 26% higher odds of new cups (OR for ASA grades 'IV + V' versus 'I' = 1.26, 95%CI 1.06-1.51, p=0.01). Patients with private versus NHS funding had six percent lower odds of receiving new stems (OR=0.94, 95%CI 0.89-0.99, p=0.02), but 13% higher odds of receiving new cups (OR=1.13, 95%CI 1.08-1.18, p<0.001).

Sensitivity analyses

Results of our first sensitivity analyses (excluding calendar-years for surgeons with <10 THRs) differed only minimally from our primary analyses (Table S8), indicating that our results were not biased by low-volume surgeons. Results of our second sensitivity analyses ('consultant in-charge' as the clustering variable) also differed only minimally from our primary analyses (Tables S9-S10).

Discussion

Sixty-eight new cups and 72 new stems were first used in THRs in the NJR for OA between 2008 and 2017. Most THRs used components introduced before 2008 but 12% used a new stem or cup. Uptake of some new implant components was very rapid. Conversely, uptake of a third of new components has been slow. Most surgeons used a maximum total of seven different cups or stems, of which one or two

For peer review only - http://bmjopen.bmj.com/site/about/guidelines.xhtml

were new components. A small number of surgeons used a wide variety of different components, including new stems, cups and combinations.

Strengths of our study include the use of the NJR dataset, the largest arthroplasty register with comprehensive data capture (>95% in the period studied). This is the first to describe the variation in factors associated with uptake of new implant components by surgeons and receipt of new components by patients. Our study has several weaknesses. We classified a component as new based on the first record of a brand name in the NJR, but this does not exclude the possibility that a component was introduced earlier to other markets outside the UK. Furthermore, new components may constitute procedures not uploaded to the NJR (missing primary THRs estimated <5%). Also, some of these components may be minor modifications or a rebadged/renamed version of an existing component and some may also cover successive versions of a component. The correct operating surgeon may not be assigned to every operation. The extent to which this applies is unknown but may result in inaccurate estimates of surgeon-level associations, although our sensitivity analyses indicate that this is unlikely. The associations we have reported may be confounded by unmeasured factors (residual confounding) and in the absence of pre-existing literature on the uptake of new implants the findings from the regression models should be considered exploratory. We were missing BMI data for some people and elected not to use multiple imputation to account for these missing data. Finally, we did not have data on hospital-level factors or regional variation in suppliers in our analyses, which may be drivers of selection.[13]

Approximately 16 new implant components/year (stems and cups) were introduced in the NJR between 2008-2017. Comparisons with Australia (34 implant components/year 2003-2008) [14] and Finland (2-4 components/year 1980-2013) [15] suggest that this rate is not unusual, but that there is large variation internationally. The rapid uptake of some new components indicates that phased introduction, as recommended in the IDEAL Framework and others,[16] are unlikely to be happening. Conversely, a third of new implant components have not yet accrued more than ten uses. Postmarket surveillance of THRs, due to their longevity, performs a safety monitoring role which cannot easily be replaced by pre-approval clinical data. Since the statistical methods are not applicable to components used in small numbers collaboration between international arthroplasty registries may allow more effective monitoring for low-volume components.

Over half of surgeons in our study used ≤5 different stems, cups, or combinations, similar to a median of two different implant brands reported by surgeons in the USA in 1997.[17] The volume of THRs

For peer review only - http://bmjopen.bmj.com/site/about/guidelines.xhtml

performed by surgeons using new components was often low (median ≤4 THRs with new components versus median 22 THRs in total), but the proportion of their THRs using any new components varied from one percent (lower quartile) to 27% (upper quartile). Surgeons who use a wider range of prosthesis combinations in THRs may have higher revision rates [18] and early THRs performed after switching implants may have a higher revision risk (a.k.a. 'learning-curve').[19] While this suggests that surgeons should rely on a narrow range of implant components and rarely switch, a phased introduction of new implant designs, as is done in Sweden, may mitigate the learning-curve effect.[20] Since there are no contemporary comparisons of the range of implant components surgeons use and their relative volumes, it is unclear whether the between-surgeon variation we have reported may be associated with worse implant survival and warrants further research.

We found that newer components were being used in patients likely to be more active (i.e. younger and/or male patients). There has been increasing evidence that uncemented implants, particularly stems, should not be used in older patients, but some uncertainty remains about their use in young patients (especially uncemented cups).[21–23] Since the majority of new cups and stems are uncemented, the decision to use these implant components in younger patients may increase the already high lifetime risk of revision surgery for these patients. Associations between BMI or ASA grade and receipt or use of new components were inconsistent between stems and cups and did not provide clear support for the use of new implant components in patients likely to be more active (i.e. lower BMI and ASA grades). It may be of interest to further investigate the implant component choices made for patients with higher BMI or ASA grades.

The most comparable previous work used NJR data to explore patient-level and hospital-level determinants that patients receive uncemented versus cemented implants.[13] Uncemented components were less likely to be used in women and older patients, and hospitals treating older patients were less likely to use them. Our results indicate that surgeons who treat a higher proportion of younger patients are more likely to use newer components. Our most marked finding, that surgeons who used a wide variety of stem-cup combinations (either established or new) were much more likely to try a new component, may be somewhat self-evident but suggests that there may be a subset of surgeons who change components more quickly than their peers. Whether this behaviour, alongside the previously discussed learning-curve, is related to outcomes of THRs is currently unclear.

For peer review only - http://bmjopen.bmj.com/site/about/guidelines.xhtml

Conclusions

A large number of new THR implant components have been introduced into use in the NJR since 2008. The majority of THRs performed since 2008 used components which have been in use for a long time, but a large number of surgeons have tried new components, with wide variation in how many types and how often they have been used. The impact of this variation on patient outcomes is currently unclear. New rather than established implant components are more likely to be used in patients who are younger and/or male, although whether this will reduce the high lifetime risk of revision for this population is unclear.

References

1 Dreinhöfer KE, Dieppe P, Stürmer T, *et al.* Indications for total hip replacement: Comparison of assessments of orthopaedic surgeons and referring physicians. *Annals of the Rheumatic Diseases* 2006;**65**:1346–50. doi:10.1136/ard.2005.047811

2 National Joint Registry for England, Wales and Northern Ireland. 14th Annual Report 2017. 2017.

3 National Institute for Health and Care Excellence. Total hip replacement and resurfacing arthroplasty for end-stage arthritis of the hip. NICE 2018.

4 Bayliss LE, Culliford D, Monk AP, *et al.* The effect of patient age at intervention on risk of implant revision after total replacement of the hip or knee: a population-based cohort study. *The Lancet* 2017;**389**:1424–30. doi:10.1016/S0140-6736(17)30059-4

5 Smith AJ, Dieppe P, Vernon K, *et al.* Failure rates of stemmed metal-on-metal hip replacements: Analysis of data from the National Joint Registry of England and Wales. *The Lancet* 2012;**379**:1199–204. doi:10.1016/S0140-6736(12)60353-5

6 de Steiger RN, Hang JR, Miller LN, *et al.* Five-Year Results of the ASR XL Acetabular System and the ASR Hip Resurfacing System. *The Journal of Bone and Joint Surgery-American Volume* 2011;**93**:2287–93. doi:10.2106/JBJS.J.01727

7 Reito A, Lehtovirta L, Lainiala O, *et al.* Lack of evidence—the anti-stepwise introduction of metal-on-metal hip replacements. *Acta Orthopaedica* 2017;**88**:478–83. doi:10.1080/17453674.2017.1353794

8 Kynaston-Pearson F, Ashmore AM, Malak TT, *et al.* Primary hip replacement prostheses and their evidence base: systematic review of literature. *BMJ* 2013;**347**:f6956. doi:10.1136/bmj.f6956

9 López-López JA, Humphriss RL, Beswick AD, *et al.* Choice of implant combinations in total hip replacement: systematic review and network meta-analysis. *BMJ (Clinical research ed)* 2017;**359**:j4651. doi:10.1136/bmj.j4651

For peer review only - http://bmjopen.bmj.com/site/about/guidelines.xhtml

10 McCulloch P, Altman DG, Campbell WB, *et al.* No surgical innovation without evaluation: the IDEAL recommendations. *The Lancet* 2009;**374**:1105–12. doi:10.1016/S0140-6736(09)61116-8

11 Birkmeyer JD, Reames BN, McCulloch P, *et al.* Understanding of regional variation in the use of surgery. *The Lancet* 2013;**382**:1121–9. doi:10.1016/S0140-6736(13)61215-5

12 National Joint Registry for England W and NI. Prostheses used in hip, knee, ankle, elbow and shoulder replacement procedures 2016. 2017.

13 Davies C. *An analysis of choice: a case study on hip prostheses*. 2011.

14 Anand R, Graves SE, de Steiger RN, *et al.* What Is the Benefit of Introducing New Hip and Knee Prostheses? *The Journal of Bone and Joint Surgery-American Volume* 2011;**93**:51–4. doi:10.2106/JBJS.K.00867

15 Peltola M. *Impact of technological change on quality of care: Studies on total hip and knee replacement*. 2016.

16 Zywiel MG, Johnson AJ, Mont MA. Graduated Introduction of Orthopaedic Implants. *The Journal of Bone & Joint Surgery* 2012;**94**:e158. doi:10.2106/JBJS.K.01675

17 Sharkey PF, Sethuraman V, Hozack WJ, *et al.* Factors influencing choice of implants in total hip arthroplasty and total knee arthroplasty: Perspectives of surgeons and patients. *Journal of Arthroplasty* 1999;**14**:281–7. doi:10.1016/S0883-5403(99)90052-9

18 Australian Orthopaedic Association. Hip, Knee & Shoulder Arthroplasty: Annual Report 2017. Australian Orthopaedic Association 2017. https://aoanjrr.sahmri.com/documents/10180/397736/Hip%2C%20Knee%20%26%20Shoulder%20 Arthroplasty

19 Peltola M, Malmivaara A, Paavola M. Hip prosthesis introduction and early revision risk. *Acta Orthopaedica* 2013;**84**:25–31. doi:10.3109/17453674.2013.771299

20 Mohaddes M, Björk M, Nemes S, *et al.* No increased risk of early revision during the implementation phase of new cup designs: Analysis of 52,903 hip arthroplasties reported to the Swedish Hip Arthroplasty Register. *Acta Orthopaedica* 2016;**87**:31–6. doi:10.1080/17453674.2016.1181818

21 Mäkelä KT, Matilainen M, Pulkkinen P, *et al.* Failure rate of cemented and uncemented total hip replacements: Register study of combined Nordic database of four nations. *BMJ (Online)* 2014;**348**:1–10. doi:10.1136/bmj.f7592

22 Stea S, Comfort T, Sedrakyan A, *et al.* Multinational comprehensive evaluation of the fixation method used in hip replacement: Interaction with age in context. *Journal of Bone and Joint Surgery - American Volume* 2014;**96**:42–51. doi:10.2106/JBJS.N.00463

23 Hailer NP, Garellick G, Kärrholm J. Uncemented and cemented primary total hip arthroplasty in the Swedish Hip Arthroplasty Register: Evaluation of 170,413 operations. *Acta Orthopaedica* 2010;**81**:34–41. doi:10.3109/17453671003685400

For peer review only - http://bmjopen.bmj.com/site/about/guidelines.xhtml

For peer review only

For peer review only - http://bmjopen.bmj.com/site/about/guidelines.xhtml

Table 1 Results from multivariable adjusted logistic regression models showing the association between surgeon-level factors and use of new stems and cups

	Stems					Cups				
**Exposure**	**Established** (n=17,753)[3]	**New** (n=2,657)[3]	**OR[1]**	**(95% CI)**	**p[1]**	**Established** (n=15,891)[3]	**New** (n=4,519)[3]	**OR[1]**	**(95% CI)**	**p[1]**
**Proportion of THRs performed on patients <55 years old**										
<10% (ref.)	12,734 (71.7%)	1,294 (48.7%)	1	-	-	11,629 (73.2%)	2,399 (53.1%)	1	-	-
≥10%	5,019 (28.3%)	1,363 (51.3%)	1.51	(1.34 to 1.69)	<0.001	4,262 (26.8%)	2,120 (46.9%)	1.45	(1.32 to 1.59)	<0.001
**Number of THRs performed in calendar year[2]** (per 10 additional cases)	7.0 (2.0, 23.0)	28.0 (9.0, 58.0)	1.02	(1.00 to 1.05)	0.03	6.0 (2.0, 20.0)	24.0 (8.0, 54.0)	1.08	(1.05 to 1.10)	<0.001
**Proportion of THRs funded privately**										
100% NHS funded (ref.)	12,551 (70.7%)	1,337 (50.3%)	1	-	-	11,420 (71.9%)	2,468 (54.6%)	1	-	-
Some or all funded privately	5,202 (29.3%)	1,320 (49.7%)	1.23	(1.06 to 1.42)	0.006	4,471 (28.1%)	2,051 (45.4%)	1.11	(0.98 to 1.25)	0.10
**Number of stem-cup combinations used in calendar year**										
≤3 (ref.)	13,915 (78.4%)	933 (35.1%)	1	-	-	12,922 (81.3%)	1,926 (42.6%)	1	-	-
4-6	3,184 (17.9%)	1,032 (38.8%)	3.97	(3.51 to 4.50)	<0.001	2,514 (15.8%)	1,702 (37.7%)	3.47	(3.12 to 3.85)	<0.001
7-10	595 (3.4%)	548 (20.6%)	9.84	(8.11 to 12.0)	<0.001	416 (2.6%)	727 (16.1%)	7.12	(5.90 to 8.61)	<0.001
>10	59 (0.3%)	144 (5.4%)	23.3	(15.3 to 35.6)	<0.001	39 (0.2%)	164 (3.6%)	13.9	(9.00 to 21.5)	<0.001

For peer review only

For peer review only - http://bmjopen.bmj.com/site/about/guidelines.xhtml

**Proportion of THRs performed on patients with ASA grade III-V**										
<25% (ref.)	12,779 (72.0%)	2,019 (76.0%)	1	-	-	11,464 (72.1%)	3,334 (73.8%)	1	-	-
≥25%	4974 (28.0%)	638 (24.0%)	1.09	(0.97 to 1.23)	0.16	4,427 (27.9%)	1,185 (26.2%)	1.23	(1.12 to 1.35)	<0.001

1 – odds ratios, 95% confidence intervals and p-values are from logistic regression models adjusted for all exposure variables, 2 - median (lower to upper quartile), 3 - proportions displayed are based on surgeon-calendar years

For peer review only

For peer review only - http://bmjopen.bmj.com/site/about/guidelines.xhtml

Table 2 Results from multivariable adjusted mixed-effects regression models (patients nested within surgeons) of age, gender, categorised BMI, ASA grade and source of funding on stem age and cup age, with category proportions

	**Stems**					**Cups**				
	**Established (n=410,613)**	**New (n=21,342)**	**OR¹**	**(95% CI)**	**p**	**Established (n=391,369)**	**New (n=40,586)**	**OR¹**	**(95% CI)**	**p**
**Age**										
<55 years old	42,078 (10.2%)	4,495 (21.1%)	2.13	(2.04 to 2.23)	<0.001	40,637 (10.4%)	5,936 (14.6%)	1.40	(1.34 to 1.45)	<0.001
55 to 80 (ref.)	306,218 (74.6%)	15,233 (71.4%)	1	-	-	292,278 (74.7%)	29,173 (71.9%)	1	-	-
≥ 80 years old	62,317 (15.2%)	1,614 (7.6%)	0.49	(0.46 to 0.52)	<0.001	58,454 (14.9%)	5,477 (13.5%)	0.90	(0.87 to 0.94)	<0.001
**Gender**										
Male (ref.)	161,920 (39.4%)	9,455 (44.3%)	1	-	-	155,709 (39.8%)	15,666 (38.6%)	1	-	-
Female	248,693 (60.6%)	11,887 (55.7%)	0.81	(0.78 to 0.84)	<0.001	235,660 (60.2%)	24,920 (61.4%)	1.11	(1.08 to 1.14)	<0.001
**BMI**										
Underweight and normal (ref.)	93,578 (22.8%)	4,639 (21.7%)	1	-	-	88,450 (22.6%)	9,767 (24.1%)	1	-	-
Overweight	162,562 (39.6%)	8,425 (39.5%)	1.04	(1.00 to 1.09)	0.06	155,070 (39.6%)	15,917 (39.2%)	0.97	(0.94 to 1.01)	0.11
Class I Obese	103,566 (25.2%)	5,495 (25.7%)	1.07	(1.02 to 1.12)	0.006	99,110 (25.3%)	9,951 (24.5%)	0.97	(0.93 to 1.00)	0.07
Class II Obese	38,175 (9.3%)	2,096 (9.8%)	1.14	(1.07 to 1.22)	<0.001	36,611 (9.4%)	3,660 (9.0%)	0.97	(0.93 to 1.02)	0.29
Class III Obese	12,732 (3.1%)	687 (3.2%)	1.07	(0.97 to 1.19)	0.17	12,128 (3.1%)	1,291 (3.2%)	1.01	(0.94 to 1.09)	0.70
**ASA grade**										
I (ref.)	58,391 (14.2%)	4,292 (20.1%)	1	-	-	55,989 (14.3%)	6,694 (16.5%)	1	-	-
II	287,661 (70.1%)	14,421 (67.6%)	0.79	(0.75 to 0.82)	<0.001	274,383 (70.1%)	27,699 (68.2%)	1.03	(0.99 to 1.06)	0.16
III	62,821 (15.3%)	2,565 (12.0%)	0.64	(0.60 to 0.69)	<0.001	59,394 (15.2%)	5,992 (14.8%)	1.08	(1.02 to 1.13)	0.003
IV + V	1,740 (0.4%)	64 (0.3%)	0.60	(0.45 to 0.80)	0.001	1,603 (0.4%)	201 (0.5%)	1.26	(1.06 to 1.51)	0.01

For peer review only - http://bmjopen.bmj.com/site/about/guidelines.xhtml

**Source of funding**										
NHS	358,057 (87.2%)	17,424 (81.6%)	1	-	-	342,458 (87.5%)	33,023 (81.4%)	1	-	-
Private	52,556 (12.8%)	3,918 (18.4%)	0.94	(0.89 to 0.99)	0.02	48,911 (12.5%)	7,563 (18.6%)	1.13	(1.08 to 1.18)	<0.001

1 – odds ratios, 95% confidence intervals and p-values are from mixed-effects logistic regression models adjusted for all exposure variables

For peer review only

For peer review only - http://bmjopen.bmj.com/site/about/guidelines.xhtml

Figure 1 Proportion of total hip replacements between January 2008 and February 2017 using stem or cups/shells introduced in different time periods (before 2004, 2004-2006, 2006-2008, 2008 onwards)

For peer review only

For peer review only - http://bmjopen.bmj.com/site/about/guidelines.xhtml

Figure 2 Cumulative total use of the top 5 new stems and cups/shells by days since they were introduced

For peer review only

For peer review only - http://bmjopen.bmj.com/site/about/guidelines.xhtml

For peer review only

For peer review only - http://bmjopen.bmj.com/site/about/guidelines.xhtml

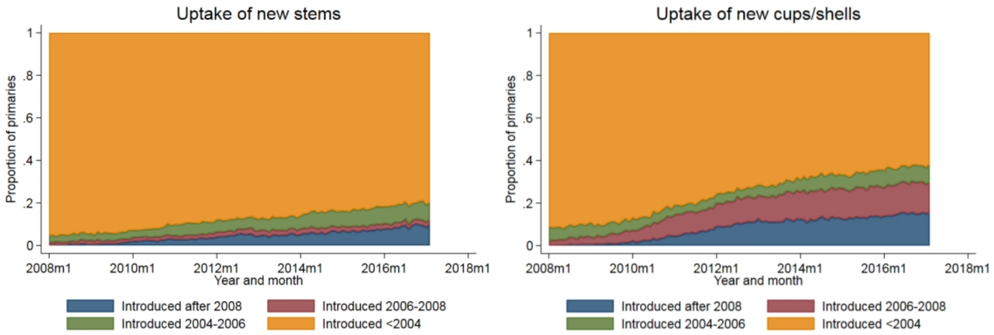

Figure 1 Proportion of total hip replacements between January 2008 and February 2017 using stem or cups/shells introduced in different time periods (before 2004, 2004-2006, 2006-2008, 2008 onwards)

640x232mm (72 x 72 DPI)

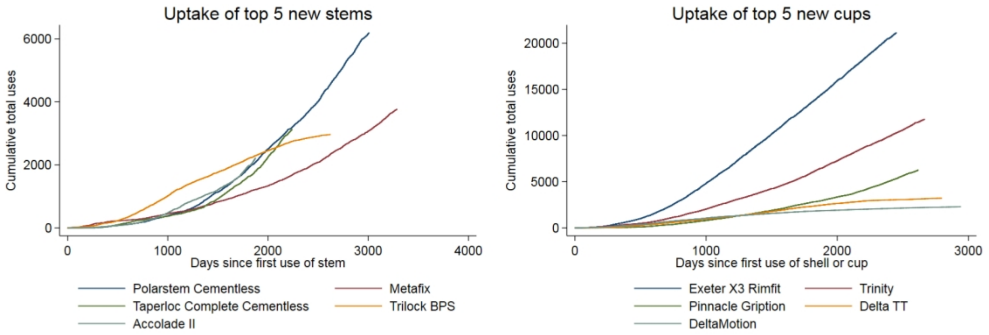

Figure 2 Cumulative total use of the top 5 new stems and cups/shells by days since they were introduced

640x232mm (72 x 72 DPI)

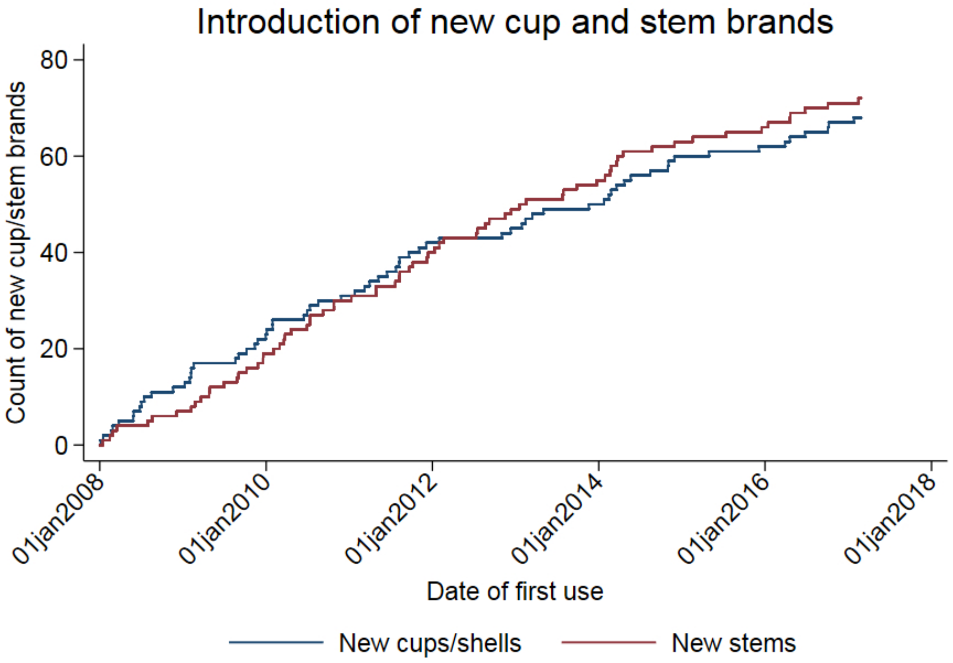

Figure S1 The cumulative introduction of new brands of cup and stem components for THRs, between January 1st 2008 and 26th February 2017

319x232mm (72 x 72 DPI)

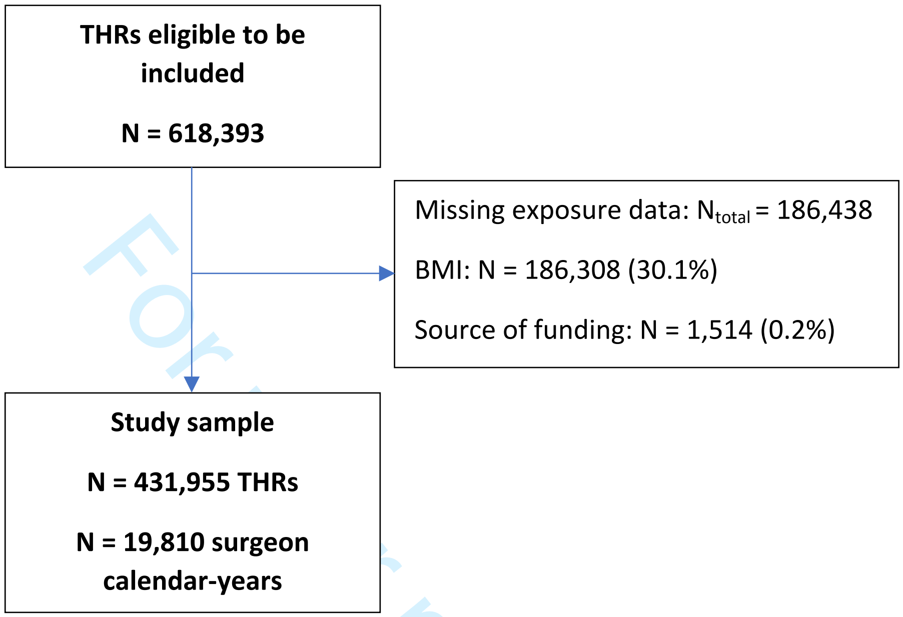

For peer review only

Supplementary material

Table S1 Uptake of new cups first used between January 1st 2008 and 26th February 2017

Cup/shell brand	UC [1]	Patients	Percent	Surgeons	Month first used	Month last used
**Exeter X3 Rimfit**		**21,116**	38.1%	**975**	**Jun 2010**	**Feb 2017**
**Trinity**	✓	**11,752**	21.2%	**310**	**Nov 2009**	**Feb 2017**
**Pinnacle Gription**	✓	**6,250**	11.3%	**599**	**Dec 2009**	**Feb 2017**
**Delta TT**	✓	**3,215**	5.8%	**193**	**Jun 2009**	**Feb 2017**
**DeltaMotion**	✓	**2,300**	4.2%	**172**	**Feb 2009**	**Feb 2017**
Versafit CC Trio	✓	1,713	3.1%	50	Mar 2011	Feb 2017
RM Pressfit Vitamys	✓	872	1.6%	37	Aug 2011	Feb 2017
Exceed ABT Cemented		865	1.6%	67	Jun 2011	Feb 2017
RM Pressfit	✓	686	1.2%	50	May 2008	Feb 2017
G7 Cementless Acetabular Component	✓	621	1.1%	36	Aug 2014	Feb 2017
AEON Cemented Acetabular Cup		614	1.1%	46	Sep 2011	Feb 2017
Plasmafit Cementless Cup	✓	546	1.0%	50	Nov 2012	Feb 2017
Allofit IT	✓	491	0.9%	21	Jan 2010	Jan 2017
Duracel		465	0.8%	45	Mar 2013	Feb 2017
ADES Cemented		342	0.6%	72	Feb 2014	Feb 2017
Regenerex Ringloc+	✓	323	0.6%	70	Feb 2009	Feb 2017
XLFit Acetabular Cup	✓	293	0.5%	56	Apr 2015	Feb 2017
April - Polyethylene	✓	220	0.4%	33	Jan 2012	Feb 2017
ADES	✓	205	0.4%	39	May 2014	Feb 2017
Delta One TT	✓	202	0.4%	82	Jun 2010	Feb 2017
Trident Constrained Cup		199	0.4%	84	Jan 2008	Feb 2017
Delta PF	✓	198	0.4%	9	Mar 2011	Nov 2014
MIHR Cup	✓	197	0.4%	12	Mar 2008	Aug 2011
Tribofit	✓	184	0.3%	10	Jul 2010	Nov 2016
seleXys TH+	✓	174	0.3%	13	Nov 2008	Apr 2011
OptiCup CEP		147	0.3%	18	Nov 2014	Feb 2017
Restoration ADM Cup	✓	140	0.3%	32	May 2011	Feb 2017
Gyros	✓	129	0.2%	28	Jan 2010	Dec 2013
Allofit-S IT	✓	126	0.2%	22	Aug 2010	Jun 2016
EcoFit Cementless Cup	✓	102	0.2%	5	Feb 2013	Mar 2015
Novation	✓	93	0.2%	10	Nov 2009	Aug 2014
M2A Magnum	✓	79	0.1%	30	Feb 2008	Jun 2010
Captiv DM	✓	78	0.1%	9	Aug 2011	Feb 2017
Freedom		78	0.1%	17	May 2008	Nov 2014
seleXys DS Cementless	✓	56	0.1%	16	Mar 2014	Dec 2016

For peer review only

Restoration Gap2		36	0.1%	24	Mar 2008	Dec 2016
MMC Resurfacing	✓	36	0.1%	10	Aug 2009	Jan 2011
ASR 300 Cup	✓	36	0.1%	1	Jan 2009	Jan 2010
MPACT	✓	30	0.1%	9	Dec 2011	Feb 2017
seleXys DS Cemented		20	0.0%	12	Feb 2014	Apr 2015
Fixa Ti-Por	✓	20	0.0%	4	Apr 2014	Oct 2016
Fixa Duplex	✓	17	0.0%	1	Mar 2016	Feb 2017
Cormet Prime	✓	12	0.0%	5	Jan 2010	Sep 2011
Delta Revision TT	✓	12	0.0%	8	Nov 2010	Aug 2016
A Class		9	0.0%	4	Feb 2009	Feb 2017
Equateur	✓	9	0.0%	5	Jul 2008	Nov 2008
U-Motion II	✓	8	0.0%	4	Apr 2016	Feb 2017
Zimmer Cemented Cup		6	0.0%	4	May 2013	Sep 2016
Regenerex Revision	✓	4	0.0%	3	Jan 2009	Jan 2012
Par-5	✓	3	0.0%	3	Jan 2008	Mar 2010
Horizon	✓	3	0.0%	2	Jul 2008	Jul 2008
2M Dual Mobility	✓	3	0.0%	2	Nov 2012	Sep 2014
Capitole C		3	0.0%	3	Jan 2013	Jul 2015
Sirius Cementless Cup	✓	3	0.0%	2	Aug 2011	Nov 2011
XPE Cup		2	0.0%	1	Jun 2016	Oct 2016
Solution Cemented Cup		2	0.0%	1	Dec 2015	Feb 2016
J-Loc	✓	2	0.0%	2	Mar 2013	Aug 2013
Evidence		2	0.0%	1	Oct 2014	Mar2015
Versafit DM	✓	2	0.0%	2	May 2008	Feb 2016
Polymax	✓	1	0.0%	1	Oct 2016	Oct 2016
Versacem		1	0.0%	1	Oct 2009	Oct 2009
Arden		1	0.0%	1	Feb 2008	Feb 2008
FIXA Duplex Cemented		1	0.0%	1	Jan 2017	Jan 2017
Endurance Cemented Cup		1	0.0%	1	Oct 2016	Oct 2016
Charnley KS		1	0.0%	1	Jul 2011	Jul 2011
Ringloc	✓	1	0.0%	1	Jan 2011	Jan 2011
Mitre Cup		1	0.0%	1	Nov 2013	Nov 2013
Capitole T	✓	1	0.0%	1	Nov 2014	Nov 2014
**Total**		**55,360**				

1 – Uncemented fixation, Rows in **bold** = five most commonly used new cups

For peer review only

Table S2 Uptake of new stems first used between January 1st 2008 and 26th February 2017

Stem brand	UC [1]	Patients	Percent	Surgeons	Month first used	Month last used
**Polarstem Cementless**	✓	**6,191**	**21.4%**	**196**	**Dec 2008**	**Feb 2017**
**Metafix Stem**	✓	**3,766**	**13.0%**	**166**	**Feb 2008**	**Feb 2017**
**Taperloc Complete Cementless Stem**	✓	**3,120**	**10.8%**	**151**	**Jan 2011**	**Feb 2017**
**Trilock BPS**	✓	**2,966**	**10.3%**	**142**	**Dec 2009**	**Feb 2017**
**Accolade II**	✓	**2,215**	**7.7%**	**180**	**Jan 2012**	**Feb 2017**
miniHip	✓	1,860	6.4%	101	Mar 2009	Feb 2017
AMIStem-H	✓	1,223	4.2%	38	Aug 2009	Jan 2017
Exeter No.1 125mm stem Line Extension		836	2.9%	211	Aug 2014	Feb 2017
Aeon Cemented Stem		724	2.5%	54	Sep 2011	Feb 2017
TriFit TS hip stem	✓	684	2.4%	44	Sep 2012	Feb 2017
SPS Evolution	✓	670	2.3%	48	Jan 2012	Feb 2017
Corail Cemented		531	1.8%	52	Apr 2009	Feb 2017
C-Stem AMT Line Extension		428	1.5%	127	Jul 2013	Feb 2017
H-Max S Monoblock Stem	✓	419	1.4%	36	May 2010	Dec 2016
EcoFit Cementless Stem	✓	319	1.1%	15	Sep 2010	Jan 2017
H-Max M Modular Stem	✓	316	1.1%	20	Mar 2010	Aug 2014
Finsbury Type C	✓	302	1.0%	39	Aug 2008	Sep 2010
Silent	✓	218	0.8%	21	Feb 2008	Feb 2014
Metha Monoblock Stem	✓	212	0.7%	26	Aug 2011	Feb 2017
Trilliance		178	0.6%	11	Jul 2011	Jan 2017
OptiStem		165	0.6%	22	Nov 2014	Feb 2017
Sirius stem		138	0.5%	10	Apr 2014	Feb 2017
Corail Revision Stem	✓	133	0.5%	89	Jul 2010	Feb 2017
Profemur L Classic	✓	132	0.5%	17	Mar 2014	Feb 2017
Profemur TL	✓	121	0.4%	23	Jan 2008	Jul 2014
AMIStem-C		110	0.4%	3	Jul 2012	Feb 2017
Master SL	✓	102	0.4%	8	Jul 2013	Feb 2017
Novation Element Stem	✓	90	0.3%	9	Nov 2009	Jul 2014
CBC Evolution	✓	83	0.3%	8	Jan 2013	Feb 2016
Nanos	✓	78	0.3%	5	Dec 2011	Nov 2016
Amoda	✓	67	0.2%	1	Apr 2010	Aug 2011
Harmony Modular	✓	65	0.2%	6	Mar 2010	Mar 2015
ABG II Cementless Stem (modular)	✓	52	0.2%	9	Apr 2009	Sep 2012
SL	✓	51	0.2%	5	Sep 2009	May 2011
XActa		47	0.2%	5	Jan 2014	Nov 2016
Avenir Muller Cementless	✓	33	0.1%	6	Jun 2016	Feb 2017
Harmony Cemented		25	0.1%	8	Feb 2014	May 2015

For peer review only

miniMax	✓	24	0.1%	2	Apr 2011	Sep 2014
SMS	✓	22	0.1%	2	Jul 2015	Oct 2016
FTS	✓	20	0.1%	5	Feb 2009	Mar 2010
Profemur TL Classic	✓	19	0.1%	5	Jan 2016	Feb 2017
Arcad Cementless	✓	17	0.1%	12	Sep 2010	Feb 2017
SMF	✓	17	0.1%	1	Oct 2011	May 2015
Echelon Cemented Stem		13	0.0%	9	Mar 2008	Dec 2016
Profemur Preserve	✓	12	0.0%	5	Feb 2012	Jun 2013
AMIStem HP	✓	12	0.0%	1	Dec 2015	May 2016
METS Cemented		12	0.0%	10	Dec 2012	Oct 2016
GMRS		11	0.0%	9	Aug 2012	Sep 2016
Harmony Cementless	✓	10	0.0%	4	Apr 2011	Apr 2015
UCP Stem		10	0.0%	5	Apr 2016	Feb 2017
Exception Cementless	✓	6	0.0%	3	Feb 2010	Mar 2010
Quadra-C		6	0.0%	2	Oct 2009	Feb 2015
Novation Stem	✓	5	0.0%	2	Mar 2014	Mar 2015
METS Cementless	✓	5	0.0%	5	Feb 2013	Feb 2016
Securus	✓	5	0.0%	5	Dec 2009	Mar 2015
G2 Cementless Stem	✓	5	0.0%	5	Dec 2013	Nov 2015
Profemur Gladiator	✓	4	0.0%	3	Mar 2010	Jul 2011
Euros Cementless	✓	3	0.0%	2	Aug 2011	Nov 2011
Atlantis	✓	3	0.0%	3	Dec 2011	Apr 2014
Restoration Cemented Stem		1	0.0%	1	Feb 2014	Feb 2014
Initiale Cemented Stem		1	0.0%	1	Jul 2008	Jul 2008
Wagner Revision Stem	✓	1	0.0%	1	Apr 2016	Apr 2016
Integrale	✓	1	0.0%	1	Jun 2009	Jun 2009
optimys	✓	1	0.0%	1	Feb 2017	Feb 2017
Prodigy	✓	1	0.0%	1	Jul 2010	Jul 2010
CDH Stem	✓	1	0.0%	1	Nov 2012	Nov 2012
Friendly		1	0.0%	1	Jul 2012	Aug 2012
Regulus Cemented Stem		1	0.0%	1	Oct 2016	Oct 2016
Arcad Cemented		1	0.0%	1	Feb 2009	Feb 2009
Endurance Cemented Stem		1	0.0%	1	Sep 2013	Sep 2013
Furlong HAC Hemiarthroplasty	✓	1	0.0%	1	Oct 2010	Oct 2010
C2 Stem	✓	1	0.0%	1	Feb 2015	Feb 2015
**Total**		**28,924**				

1 – Uncemented fixation, Rows in **bold** = five most commonly used new stems

Table S3 Number of different post-2008 shells/cups used by surgeons

Number of new cups used	Number of surgeons	Percent	Cumulative percent
1	1,280	61.9%	61.9%
2	457	22.1%	84.0%
3	189	9.1%	93.1%
4	84	4.1%	97.2%
5	31	1.5%	98.7%
6	16	0.8%	99.4%
7	7	0.3%	99.8%
8	4	0.2%	100.0%
11	1	0.1%	100.0%
**Total**	2,069		

For peer review only

Table S4 Number of different post-2008 stems used by surgeons

Number of new stems used	Number of surgeons	Percent	Cumulative percent
1	888	67.6%	67.6%
2	260	19.8%	87.4%
3	97	7.4%	94.8%
4	38	2.9%	97.7%
5	15	1.1%	98.9%
6	9	0.7%	99.5%
7	4	0.3%	99.9%
8	1	0.1%	99.9%
9	1	0.1%	100.0%
**Total**	1,313		

For peer review only

Table S5 The number of unique new stem-cup combinations used simultaneously by surgeons

Stem-cup combinations	Number of surgeons	Percent	Cumulative percent
1	437	70.0%	70.0%
2	113	18.1%	88.1%
3	41	6.6%	94.7%
4	17	2.7%	97.4%
5	6	1.0%	98.4%
6	2	0.3%	98.7%
7	2	0.3%	99.0%
8	3	0.5%	99.5%
9	2	0.3%	99.8%
13	1	0.2%	100.%
**Total**	624	100%	

For peer review only

Table S6 Results from unadjusted logistic regression models showing the association between surgeon-level factors and use of new versus old stems and cups

	Stems			Cups		
**Exposure**	**OR[1]**	**(95% CI)**	**p**	**OR[1]**	**(95% CI)**	**p**
**Proportion of THRs performed on patients <55 years old**						
<10% (ref.)	1		-	1		-
≥10%	2.67	(2.39 to 2.98)	<0.001	2.41	(2.20 to 2.64)	<0.001
**Number of THRs performed in calendar year[2]** (per 10 additional cases)	1.19	(1.16 to 1.22)	<0.001	1.23	(1.20 to 1.26)	<0.001
**Proportion of THRs funded privately**						
100% NHS funded (ref.)	1		-	1		-
Some or all funded privately	2.38	(2.09 to 2.71)	<0.001	2.12	(1.91 to 2.36)	<0.001
**Number of stem-cup combinations used in calendar year**						
≤3 (ref.)	1		-	1		-
4-6	4.83	(4.31 to 5.42)	<0.001	4.54	(4.13 to 5.00)	<0.001
7-10	13.7	(11.6 to 16.3)	<0.001	11.7	(9.91 to 13.9)	<0.001
>10	36.4	(24.4 to 54.4)	<0.001	28.2	(18.4 to 43.3)	<0.001
**Proportion of THRs performed on patients with ASA grade III-V**						
<25% (ref.)	1		-	1		-
≥25%	0.81	(0.72 to 0.91)	0.001	0.92	(0.84 to 1.01)	0.08

1 – odds ratios, 95% confidence intervals and p-values are from unadjusted logistic regression models

For peer review only

Table S7 Results from unadjusted mixed-effects regression models (patients nested within surgeons) of age, gender, categorised BMI, ASA grade, and source of funding on stem and cup age

	**Stems**			**Cups**		
	**OR[1]**	**(95% CI)**	**p**	**OR[1]**	**(95% CI)**	**p**
**Age** (years)						
<55 years old	2.31	(2.21 to 2.42)	<0.001	1.37	(1.32 to 1.42)	<0.001
55 to 80 (ref.)	1	-	-	1	-	-
≥ 80 years old	0.45	(0.42 to 0.48)	<0.001	0.92	(0.89 to 0.96)	<0.001
**Gender**						
Male (ref.)	1	-	-	1	-	-
Female	0.77	(0.75 to 0.80)	<0.001	1.10	(1.07 to 1.13)	<0.001
**BMI**						
Underweight and normal (ref.)	1	-	-	1	-	-
Overweight	1.11	(1.06 to 1.16)	<0.001	0.96	(0.93 to 0.99)	0.01
Class I Obese	1.16	(1.11 to 1.22)	<0.001	0.96	(0.93 to 1.00)	0.04
Class II Obese	1.25	(1.18 to 1.33)	<0.001	1.00	(0.95 to 1.05)	0.90
Class III Obese	1.18	(1.07 to 1.30)	0.001	1.07	(1.00 to 1.15)	0.07
**ASA grade**						
I (ref.)	1	-	-	1	-	-
II	0.64	(0.61 to 0.67)	<0.001	0.95	(0.92 to 0.99)	0.007
III	0.47	(0.44 to 0.50)	<0.001	0.96	(0.92 to 1.01)	0.13
IV + V	0.40	(0.30 to 0.53)	<0.001	1.10	(0.93 to 1.33)	0.24
**Source of funding**						
NHS	1	-	-	1	-	-
Private	0.93	(0.88 to 0.98)	0.005	1.11	(1.06 to 1.16)	<0.001

1 – odds ratios, 95% confidence intervals and p-values are from unadjusted mixed-effects logistic regression models

For peer review only

Table S8 Sensitivity analysis 1: Results from multivariable adjusted logistic regression models showing the association between surgeon-level factors and use of new versus old stems and cups, excluding surgeon calendar-years with <10 THRs

	Stems			Cups		
**Exposure**	**OR[1]**	**(95% CI)**	**p**	**OR[1]**	**(95% CI)**	**p**
**Proportion of THRs performed on patients <55 years old**						
<10% (ref.)	1	-	-	1	-	-
≥10%	1.30	(1.14 to 1.50)	<0.001	1.42	(1.26 to 1.59)	<0.001
**Number of THRs performed in calendar year[2]** (per 10 additional cases)	1.02	(1.00 to 1.05)	0.04	1.05	(1.03 to 1.08)	<0.001
**Proportion of THRs funded privately**						
100% NHS funded (ref.)	1	-	-	1	-	-
Some or all funded privately	1.27	(1.08 to 1.49)	0.004	1.09	(0.95 to 1.25)	0.22
**Number of stem-cup combinations used in calendar year**						
≤3 (ref.)	1	-	-	1	-	-
4-6	3.57	(3.04 to 4.18)	<0.001	3.05	(2.69 to 3.46)	<0.001
7-10	9.24	(7.47 to 11.4)	<0.001	6.45	(5.30 to 7.86)	<0.001
>10	22.1	(14.4 to 33.8)	<0.001	13.1	(8.48 to 20.4)	<0.001
**Proportion of THRs performed on patients with ASA grade III-V**						
<25% (ref.)	1	-	-	1	-	-
≥25%	1.20	(1.03 to 1.41)	0.02	1.32	(1.16 to 1.50)	<0.001

1 – odds ratios, 95% confidence intervals and p-values are from logistic regression models adjusted for all exposure variables

For peer review only

Table S9 Sensitivity analysis 2a: Results from multivariable adjusted logistic regression models showing the association between surgeon-level factors (consultant in-charge) and use of new versus old stems and cups

	Stems			Cups		
**Exposure**	**OR[1]**	**(95% CI)**	**p**	**OR[1]**	**(95% CI)**	**p**
**Proportion of THRs performed on patients <55 years old**						
<10% (ref.)	1	-	-	1	-	-
≥10%	1.49	(1.31 to 1.70)	<0.001	1.57	(1.41 to 1.75)	<0.001
**Number of THRs performed in calendar year[2]** (per 10 additional cases)	1.03	(1.01 to 1.04)	0.003	1.07	(1.05 to 1.09)	<0.001
**Proportion of THRs funded privately**						
100% NHS funded (ref.)	1	-	-	1	-	-
Some or all funded privately	1.27	(1.09 to 1.49)	0.002	1.17	(1.02 to 1.33)	0.02
**Number of stem-cup combinations used in calendar year**						
≤3 (ref.)	1	-	-	1	-	-
4-6	3.69	(3.19 to 4.27)	<0.001	3.11	(2.76 to 3.53)	<0.001
7-10	8.78	(7.9 to 10.9)	<0.001	5.61	(4.58 to 6.88)	<0.001
>10	22.9	(15.2 to 34.6)	<0.001	11.4	(7.46 to 17.3)	<0.001
**Proportion of THRs performed on patients with ASA grade III-V**						
<25% (ref.)	1	-	-	1	-	-
≥25%	1.22	(1.06 to 1.42)	0.007	1.25	(1.11 to 1.41)	<0.001

1 – odds ratios, 95% confidence intervals and p-values are from logistic regression models adjusted for all exposure variables

For peer review only

Table S10 Sensitivity analysis 2b: Results from multivariable adjusted mixed-effects regression models (patients nested within 'consultant in-charge') of age, gender, categorised BMI, ASA grade, and source of funding on stem and cup age

	Stems			Cups		
	**OR[1]**	**(95% CI)**	**p**	**OR[1]**	**(95% CI)**	**p**
**Age** (years)						
<55 years old	2.20	(2.10 to 2.30)	<0.001	1.40	(1.35 to 1.46)	<0.001
55 to 80 (ref.)	1	-	-	1	-	-
≥ 80 years old	0.48	(0.45 to 0.51)	<0.001	0.91	(0.88 to 0.95)	<0.001
**Gender**						
Male (ref.)	1	-	-	1	-	-
Female	0.81	(0.78 to 0.84)	<0.001	1.11	(1.08 to 1.14)	<0.001
**BMI**						
Underweight and normal (ref.)	1	-	-	1	-	-
Overweight	1.03	(0.99 to 1.08)	0.18	0.97	(0.94 to 1.00)	0.08
Class I Obese	1.05	(1.00 to 1.11)	0.03	0.96	(0.92 to 0.99)	0.02
Class II Obese	1.12	(1.05 to 1.20)	0.001	0.98	(0.93 to 1.03)	0.37
Class III Obese	1.07	(0.96 to 1.18)	0.21	1.02	(0.95 to 1.10)	0.53
**ASA grade**						
I (ref.)	1	-	-	1	-	-
II	0.77	(0.74 to 0.81)	<0.001	1.02	(0.98 to 1.05)	0.41
III	0.62	(0.58 to 0.67)	<0.001	1.05	(1.00 to 1.10)	0.04
IV + V	0.59	(0.44 to 0.79)	<0.001	1.20	(1.00 to 1.43)	0.05
**Source of funding**						
NHS	1	-	-	1	-	-
Private	1.05	(1.00 to 1.11)	0.06	1.16	(1.12 to 1.21)	<0.001

1 – odds ratios, 95% confidence intervals and p-values are from mixed-effects logistic regression models adjusted for all exposure variables

For peer review only

Figure S1 The cumulative introduction of new brands of cup and stem components for THRs, between January 1st 2008 and 26th February 2017

For peer review only

Figure S2 STROBE Flow diagram

For peer review only

Reporting checklist for cohort study.

Based on the STROBE cohort guidelines.

Instructions to authors

Complete this checklist by entering the page numbers from your manuscript where readers will find each of the items listed below.

Your article may not currently address all the items on the checklist. Please modify your text to include the missing information. If you are certain that an item does not apply, please write "n/a" and provide a short explanation.

Upload your completed checklist as an extra file when you submit to a journal.

In your methods section, say that you used the STROBE cohort reporting guidelines, and cite them as:

von Elm E, Altman DG, Egger M, Pocock SJ, Gotzsche PC, Vandenbroucke JP. The Strengthening the Reporting of Observational Studies in Epidemiology (STROBE) Statement: guidelines for reporting observational studies.

For peer review only

		Reporting Item	Page Number
Title	#1a	Indicate the study’s design with a commonly used term in the title or the abstract	4
Abstract	#1b	Provide in the abstract an informative and balanced summary of what was done and what was found	4
Background / rationale	#2	Explain the scientific background and rationale for the investigation being reported	6
Objectives	#3	State specific objectives, including any prespecified hypotheses	6
Study design	#4	Present key elements of study design early in the paper	7
Setting	#5	Describe the setting, locations, and relevant dates, including periods of recruitment, exposure, follow-up, and data collection	7
Eligibility criteria	#6a	Give the eligibility criteria, and the sources and methods of selection of participants. Describe methods of follow-up.	7
	#6b	For matched studies, give matching criteria and number of exposed and	See note

		unexposed	1
Variables	#7	Clearly define all outcomes, exposures, predictors, potential confounders, and effect modifiers. Give diagnostic criteria, if applicable	7-8
Data sources / measurement	#8	For each variable of interest give sources of data and details of methods of assessment (measurement). Describe comparability of assessment methods if there is more than one group. Give information separately for for exposed and unexposed groups if applicable.	7-8
Bias	#9	Describe any efforts to address potential sources of bias	8-9
Study size	#10	Explain how the study size was arrived at	See note 2
Quantitative variables	#11	Explain how quantitative variables were handled in the analyses. If applicable, describe which groupings were chosen, and why	8-9
Statistical methods	#12a	Describe all statistical methods, including those used to control for confounding	8-9
	#12b	Describe any methods used to examine subgroups and interactions	8-9
	#12c	Explain how missing data were addressed	8
	#12d	If applicable, explain how loss to follow-up was addressed	See note 3
	#12e	Describe any sensitivity analyses	8-9
Participants	#13a	Report numbers of individuals at each stage of study—eg numbers potentially eligible, examined for eligibility, confirmed eligible, included in the study, completing follow-up, and analysed. Give information separately for for exposed and unexposed groups if applicable.	9-10
	#13b	Give reasons for non-participation at each stage	9-10
	#13c	Consider use of a flow diagram	Figure S2
Descriptive data	#14a	Give characteristics of study participants (eg demographic, clinical, social) and information on exposures and potential confounders. Give information separately for exposed and unexposed groups if applicable.	9-10
	#14b	Indicate number of participants with missing data for each variable of	See note

For peer review only

		interest	4
	#14c	Summarise follow-up time (eg, average and total amount)	See note 5
Outcome data	#15	Report numbers of outcome events or summary measures over time. Give information separately for exposed and unexposed groups if applicable.	See note 6
Main results	#16a	Give unadjusted estimates and, if applicable, confounder-adjusted estimates and their precision (eg, 95% confidence interval). Make clear which confounders were adjusted for and why they were included	See note 7
	#16b	Report category boundaries when continuous variables were categorized	See note 8
	#16c	If relevant, consider translating estimates of relative risk into absolute risk for a meaningful time period	See note 9
Other analyses	#17	Report other analyses done—e.g., analyses of subgroups and interactions, and sensitivity analyses	9-11
Key results	#18	Summarise key results with reference to study objectives	11-12
Limitations	#19	Discuss limitations of the study, taking into account sources of potential bias or imprecision. Discuss both direction and magnitude of any potential bias.	12
Interpretation	#20	Give a cautious overall interpretation considering objectives, limitations, multiplicity of analyses, results from similar studies, and other relevant evidence.	12-13
Generalisability	#21	Discuss the generalisability (external validity) of the study results	14
Funding	#22	Give the source of funding and the role of the funders for the present study and, if applicable, for the original study on which the present article is based	3

Author notes

1. n/a - not relevant

2. n/a - not relevant

3. n/a - not relevant

4. 10 and Fig S2

For peer review only

5. n/a - not relevant

6. Tables 1 & 2

7. Tables S6 & S7

8. n/a - not needed

9. n/a - not needed

The STROBE checklist is distributed under the terms of the Creative Commons Attribution License CC-BY.
This checklist was completed on 31. January 2019 using https://www.goodreports.org/, a tool made by the EQUATOR Network in collaboration with Penelope.ai

For peer review only

BMJ Open

Understanding the uptake of new hip replacement implants in the UK: A cohort study using data from the National Joint Registry for England and Wales

Journal:	*BMJ Open*
Manuscript ID	bmjopen-2019-029572.R1
Article Type:	Original research
Date Submitted by the Author:	03-Sep-2019
Complete List of Authors:	Penfold, Chris; University of Bristol, Musculoskeletal Research Unit, Translational Health Sciences, Bristol Medical School; University Hospitals Bristol NHS Foundation Trust and University of Bristol, National Institute for Health Research Bristol Biomedical Research Centre<br>Blom, AW; University of Bristol, Musculoskeletal Research Unit, Translational Health Sciences, Bristol Medical School; University Hospitals Bristol NHS Foundation Trust and University of Bristol, National Institute for Health Research Bristol Biomedical Research Centre<br>Sayers, Adrian; University of Bristol, Muscloskeletal Research Unit, Translational Health Sciences, Bristol Medical School; University of Bristol, Population Health Sciences, Bristol Medical School<br>Wilkinson, J. Mark; Northern General Hospital, Metabolic Bone Unit, Sorby Wing; University of Sheffield, 5. Department of Oncology and Metabolism and The Mellanby Centre for Bone Research<br>Hunt, Linda; University of Bristol, Musculoskeletal Research Unit, Translational Health Sciences, Bristol Medical School<br>Judge, Andrew; University of Bristol, Musculoskeletal Research Unit, Translational Health Sciences, Bristol Medical School; University Hospitals Bristol NHS Foundation Trust and University of Bristol, National Institute for Health Research Bristol Biomedical Research Centre<br>Whitehouse, Michael; University of Bristol, Musculoskeletal Research Unit, Translational Health Sciences, Bristol Medical School; University Hospitals Bristol NHS Foundation Trust and University of Bristol, National Institute for Health Research Bristol Biomedical Research Centre
**Primary Subject Heading**:	Surgery
Secondary Subject Heading:	Health services research
Keywords:	Orthopaedic & trauma surgery < SURGERY, Joint replacement, Implant, Patient, Surgeon, National Joint Registry

SCHOLARONE™
Manuscripts

Understanding the uptake of new hip replacement implants in the UK: A cohort study using data from the National Joint Registry for England and Wales

Chris M Penfold[1+2*]

Ashley W Blom[1+2]

Adrian Sayers[1+3]

J Mark Wilkinson[4+5]

Linda P Hunt[1]

Andrew Judge[1+2]

Michael R Whitehouse[1+2]

Affiliations:

1. Musculoskeletal Research Unit, Translational Health Sciences, Bristol Medical School, 1st Floor Learning & Research Building, Southmead Hospital, Bristol, BS10 5NB, UK
2. National Institute for Health Research Bristol Biomedical Research Centre, University Hospitals Bristol NHS Foundation Trust and University of Bristol.
3. Population Health Sciences, Bristol Medical School, University of Bristol, Bristol
4. Metabolic Bone Unit, Sorby Wing, Northern General Hospital, Sheffield, S5 7AU, UK
5. Department of Oncology and Metabolism and The Mellanby Centre for Bone Research, University of Sheffield

For peer review only

For peer review only - http://bmjopen.bmj.com/site/about/guidelines.xhtml

For peer review only

For peer review only - http://bmjopen.bmj.com/site/about/guidelines.xhtml

* - denotes corresponding author

Corresponding author contact details:

Email: chris.penfold@bristol.ac.uk

Address: Musculoskeletal Research Unit, University of Bristol, School of Clinical Sciences, Learning and Research Building, Southmead Hospital, Bristol, BS10 5NB

Telephone: 0117 41 47863

For peer review only

Keywords

Orthopaedics, joint replacement, implant, patient, surgeon, national joint registry

Data sharing statement

Access to the data analysed in this study required permission from the National Joint Registry for England, Wales and Northern Ireland Research Sub-committee. http://www.njrcentre.org.uk/njrcentre/Research/Researchrequests/tabid/305/Default.aspx contains information on research data access request to the National Joint Registry.

Acknowledgements

This study was funded by the NIHR Biomedical Research Centre at University Hospitals Bristol NHS Foundation Trust and the University of Bristol. The views expressed are those of the author(s) and not necessarily those of the NIHR or the Department of Health and Social Care. We thank the patients and staff of all the hospitals who have contributed data to the National Joint Registry. We are grateful to the Healthcare Quality Improvement Partnership, the National Joint Registry Steering Committee, and staff at the National Joint Registry for facilitating this work.

For peer review only - http://bmjopen.bmj.com/site/about/guidelines.xhtml

Disclaimer

The views expressed represent those of the authors and do not necessarily reflect those of the National Joint Registry Steering Committee or Healthcare Quality Improvement Partnership, who do not vouch for how the information is presented. The views expressed in this article are those of the authors and not necessarily those of the NHS, the NIHR, or the Department of Health and Social Care.

Ethics approval

Patient consent was obtained for data collection by the National Joint Registry. According to the specifications of the NHS Health Research Authority, separate informed consent and ethical approval were not required for the present study.

Author Contributions

CP, AB, AJ and MW designed the study. CP, AB, AS, JMW, LH, AJ and MW reviewed the published work. CP conducted the statistical analysis and wrote the manuscript. All authors contributed to critical review of the manuscript and its revision. CP had full access to all the data and AB is the guarantor.

Conflicts of Interest

Competing interests: All authors have completed the ICMJE uniform disclosure form at www.icmje.org/coi_disclosure.pdf and declare: no support from any organisation for the submitted work. AB and MW are involved in a separate grant to the University of Bristol funded by Stryker. All other authors declare no financial relationships with any organisations that might have an interest in the submitted work in the previous three years. All authors declare no other relationships or activities that could appear to have influenced the submitted work.

For peer review only

For peer review only - http://bmjopen.bmj.com/site/about/guidelines.xhtml

Funding

This study was funded by the NIHR Biomedical Research Centre at University Hospitals Bristol NHS Foundation Trust and the University of Bristol. Adrian Sayers was supported by a MRC fellowship MR/L01226X/1.

Word count

4055 words

Abstract

Objectives

Primary: describe uptake of new implant components (femoral stem or acetabular cup/shell) for total hip replacements (THRs) in the National Joint Registry for England and Wales (NJR). Secondary: compare the characteristics of: a) surgeons b) patients who used/received new rather than established components.

Design

Cohort of 618,393 primary THRs performed for osteoarthritis (±other indications) by 4,979 surgeons between 2008-2017 in England and Wales from the NJR. We described the uptake of new (first recorded use >2008, used within 5 years) stems/cups, and variation in uptake by surgeons (primary objectives). We explored surgeon-level and patient-level factors associated with use/receipt of new components with logistic regression models (secondary objectives).

Outcomes

Primary outcomes: total number of new cups/stems, proportion of operations using new versus established components. Secondary outcomes: odds of: a) a surgeon using a new cup/stem in a calendar-year, b) a patient receiving a new rather than established cup/stem.

Results

Sixty-eight new cups and 72 new stems were used in 47,606 primary THRs (7.7%) by 2,005 surgeons (40.3%) 2008-2017. Surgeons used a median of one new stem and cup (25%-75%=1-2 both, max=10

For peer review only - http://bmjopen.bmj.com/site/about/guidelines.xhtml

For peer review only

cups, max=8 stems). Surgeons performed a median total of 22 THRs (25%-75%=5-124, range=1-3,938) in the period 2008-2017. Surgeons used new stems in a median of 5.0% (25%-75%=1.3-16.1%) and new cups in a median of 9.4% (25%-75%=2.8-26.7%) of their THRs. Patients aged <55 years old versus those 55-80 had higher odds of receiving a new rather than established stem (OR=1.83, 95%CI=1.73-1.93) and cup (OR=1.31, 95%CI=1.25-1.37). Women had lower odds of receiving a new stem (OR=0.87, 95%CI=0.84-0.90), higher odds of receiving a new cup (OR=1.06, 95%CI=1.03-1.09).

Conclusions

Large numbers of new THR components have been introduced in the NJR since 2008. 40% of surgeons have tried new components, with wide variation in how many types and frequency they have been used.

Article Summary

Strengths and limitations of this study

- This study provides a nationally representative description of the uptake of new implant components for total hip replacements in England and Wales.
- This is the first study to describe the variation in uptake of new components by surgeons, and surgeon characteristics which may be associated with the use of new components.
- Although implant component brand names were checked by the authors, some components may have been reclassified or we may still have misclassified some components as either new or established, but the introduction of unique device identifiers should remove this problem in future.
- The surgeon assigned as lead operating surgeon in the NJR may not be correct, although consistency between our sensitivity and primary analyses indicate that this is unlikely to have substantially affected our findings.
- Hospital-level or regional variation in suppliers may be important factors affecting implant uptake, but these were beyond the scope of this study.

For peer review only - http://bmjopen.bmj.com/site/about/guidelines.xhtml

For peer review only

Background

Total hip replacements (THRs) are mainly performed to treat pain and functional limitation due to osteoarthritis (OA).[1] It is a highly successful surgical procedure with typical 10-year revision rates <5%,[2] the current NICE benchmark.[3] However, younger patients are more likely to require revision surgery; the lifetime revision risk for men having a THR in their 50s is ~35% compared with 5% in their 70s.[4] Such patients may benefit the most from developments in THR that lead to reduced revision rates or improved outcomes. However, they may also be affected for the longest time if these developments lead to poorer outcomes.

Some new implant designs intended to benefit these more active and/or younger patients have been high-profile failures, for example metal-on-metal THRs [5] including the Articular Surface Replacement (ASR) prostheses in particular.[6] Many new implants, the ASR included,[7] were introduced with minimal supporting evidence of their effectiveness [8] and may offer at best no improvement over pre-existing components.[9] An influential agenda for surgery research (IDEAL) was developed, providing a framework for future investigations into surgical innovations, which recommended the phased introduction of new medical devices.[10] The rapid uptake of ASR hip replacements before the publication of supporting evidence bypassed IDEAL Stages 2a (‘Development’) and 2b (‘Early dispersion and exploration’). Instead, long-term monitoring was relied on to monitor outcomes (Stage 4).[7] It is not clear whether the uptake of newer implants has also been rapid.

There is wide variation between and within regions in the use of common surgical procedures, which are only explained to a small degree by differing patient demands and diagnostic practices.[11] The large number of different components used in primary THRs (127 femoral stems and 105 acetabular cups recorded in the National Joint Registry for England, Wales, Northern Ireland and the Isle of Man (NJR) in 2016) [12] may be an important source of variation. Many registries describe the volume of different implant components used annually but not the variation in uptake of new implants between surgeons or which patients receive them. More research is needed to understand and reduce avoidable variation in outcomes created by differences in surgical activity.

We aimed to:

1. Describe the uptake of new implants for THRs in the NJR
2. Describe how this uptake varies by surgeons
3. Compare surgeons who use new compared with established components

For peer review only - http://bmjopen.bmj.com/site/about/guidelines.xhtml

4. Compare patients who receive new compared with established components

Methods

Data Source

The NJR was established in 2003.[2] Data entry for Northern Ireland and the Isle of Man did not commence until 2013 and 2015, respectively therefore they are excluded from this analysis. Key markers of NJR data quality were high and stable from 2008 onwards [13].

Study sample

We included the cohort of patients who received a primary THR for OA (± other indications) between 1st January 2008 and 26th February 2017. We used NJR data from 2003 onwards to calculate the date each implant component was first used and the total number of implantations. We excluded people who had not given consent for recording of personal details, where the brand of their acetabular or femoral components was uncertain, and those who received a resurfacing rather than stemmed THR. Resurfacing THRs were excluded since patients who receive these are a very different demographic from those receiving stemmed THRs (significantly younger and more likely to be male), and the annual volume is very low (~550 in 2017) and decreasing [14].

Patient involvement

This study was designed and undertaken without patient involvement.

Definition of new and established implant components

We identified the implant component brand from component labels recorded in the NJR. We used the earliest recorded use by any surgeon in the NJR of each femoral (stem) or acetabular (cup or shell) component to define an implant component's start date. We classified implant components with a start date between the beginning of NJR data collection (2003) and the end of 2007 as 'established'. This allowed implant components which were in use before the NJR started but which may have only been used occasionally to be recorded in the NJR and classified appropriately as 'established'. NJR data quality was also high and stable from 2008 onwards. Implant components with a start date on or after 1st

For peer review only - http://bmjopen.bmj.com/site/about/guidelines.xhtml

For peer review only

January 2008 and which were used within five years of this start date were classified as ‘new’. Those used later than five years after their start date were classified as ‘established’.

Surgeon uptake of new implant components

All surgeons with operations recorded in the NJR are assigned an anonymised identifier and their role in the operation (“consultant in charge” or “operating”) is recorded. We summarised each operating surgeon’s activity across each calendar-year in which they performed ≥1 THR. We considered five potential surgeon-level factors which may be associated with use of a new component in a calendar-year: total volume of THRs performed in that year, proportion of those THRs performed on patients <55 years old (<10% and ≥10%), source of funding for THRs (‘100% NHS funded’ or ‘some or all privately funded’), proportion of THRs performed on patients with an American Society of Anaesthesiologists (ASA) grade III-V (<25% and ≥25%), and the range of different stem-cup combinations used in that calendar-year (‘≤3’, ‘4-6’, ‘7-10’ and ‘>10’). Surgeons who performed ≥10% of their THRs on patients aged <55 years old and those who performed ≥25% of their THRs on patients with ASA III-V were in approximately the upper quartile of these distributions.

Patients receiving new implant components

We used date of surgery to order patients within implant components and within surgeons. We categorised patients according to whether the component they received was new or established. We considered five potential patient-level factors which may be associated with their receipt of new components: age at the time of THR (<55, 55-80, and 80+ years), gender, body mass index (BMI), ASA grade, and NHS or private funding. We selected these categories for age to reflect patients who were having a primary THR at a relatively young or relatively old age, the median age at the time of primary THR was 69 years (25%-75% 61-76 years).[14]

Statistical analyses

We described the use of unique stems and cups in primary THRs performed since January 1st 2008, the cumulative use of new components in patients, and the count of surgeons who used new components. We also described the total number of all and new cups, stems, and combinations.

For peer review only - http://bmjopen.bmj.com/site/about/guidelines.xhtml

Surgeon-level factors

In analyses of surgeon-level and patient-level factors associated with use of or receipt of new implants we included only those people with complete exposure and outcome data for the surgeon-level and patient-level analysis models (i.e. complete case analysis). We assumed that data were missing at random but did not use multiple imputation to account for these missing data since there were no variables in the NJR dataset which were not already in our regression models and which may have carried information about the missing data (particularly BMI).

Our outcome was whether a surgeon used a new component at least once for a THR in a calendar-year (stems and cups analysed separately), unit of analysis was surgeon calendar-years and exposure variables were those surgeon-level factors defined previously. We used multivariable logistic regression models, accounting for the clustering of calendar-years within surgeons.

Patient-level factors

Our outcome was whether a patient received a new rather than established component (stems and cups analysed separately), unit of analysis was patients and exposure variables were those patient-level factors defined previously. Patient-level factors were included in multivariable mixed-effects logistic regression models, with patients nested within surgeons.

Sensitivity analyses

We conducted three sensitivity analyses. To determine whether the lack of variability in patients operated on by low volume surgeons affected our results we repeated our surgeon-level analysis excluding calendar-years for surgeons in which they performed <10 THRs. We also considered that the choice of component was made by the consultant in-charge rather than the operating surgeon (the consultant in-charge was not the operating surgeon for ~16% of THRs). We repeated our surgeon-level analysis by consultant in-charge and repeated our patient-level analysis with patients clustered within consultant in-charge.

In order to determine the extent to which patients with complete data for all exposures and outcome variables differed from those missing some exposure data (mainly BMI) we compared these groups using chi-square tests. We also repeated our patient-level analyses for those patients with complete data for all exposure variables (including BMI) but excluding BMI from the model, and for those with complete data for all exposure variable (excluding BMI).

For peer review only - http://bmjopen.bmj.com/site/about/guidelines.xhtml

All analyses were performed using Stata v15 (StataCorp).

Results

Overall use of implant components

Between 1st January 2008 and 26th February 2017, 618,393 primary THRs were performed for OA in England and Wales and recorded in the NJR, corresponding to 23,887 calendar-years in which surgeons performed ≥1 THR. The mean age of the patients was 68.5 years (SD=11.1 years), 60.7% were female, their ASA grades were I:14.2%, II:69.9%, III:15.5% and IV/V:0.5%. Twenty-three percent had a normal/underweight BMI, 39.6% were overweight and 37.6% obese. THRs were performed by 4,979 surgeons using 189 different stems, 187 cups and 2,026 stem-cup combinations. Surgeons used a median of three different stems (25%-75%=2-5, max=21), four cups (25%-75%=2-7, max=27) and five combinations (25%-75%=2-9, max=60). They performed a median total of 22 THRs between 2008 and 2017 (25%-75%=5-124, range 1-3,938), although this includes surgeons who started part way through this period, retired or changed their practice. Excluding calendar-years in which a surgeon performed no THRs, the median number of THRs surgeons performed per year was 11 (25%-75%=3-35, range 1-584) and in 47% of surgeon calendar-years (11,164 of 23,887) surgeons performed <10 THRs.

Use of new implant components

During this period 68 new cups (47 uncemented, Table S1) and 72 new stems (51 uncemented, Table S2) were first used. The rate of introduction of new cups and stems remained stable (~16 new components/year, Figure S1). Eight percent (n= 47,606) of THRs performed used a new stem, cup, or combination. Forty percent (n=2,005) of surgeons who performed a THR in this period used at least one new implant component.

New cups were used in 5.8% (n= 35,885) THRs performed by 34.1% (n=1,699) surgeons (Table S1), new stems in 2.9% (n= 18,159) THRs by 22.3% (n=1,111) surgeons (Table S2) and new combinations in 1.0% (n= 6,438) THRs by 8.7% (n=433) surgeons. Most new cups (n= 19,775, 55.1%) and almost all new stems (n= 15,361, 84.6%) were uncemented. The median number of new stems, cups and combinations used by surgeons was one (25%-75%=1-2, cups max=10, stems max=8 and combinations max=9; Table S3, Table S4 & Table S5). The median THRs performed using new stems was three (25%-75%=1-11, max=637) and new cups was three (25%-75%=1-14, max=867). The median proportion of a surgeon's

For peer review only - http://bmjopen.bmj.com/site/about/guidelines.xhtml

THRs performed using new stems was 3.4% (25%-75%=1.0-10.6%), new cups 6.3% (25%-75%=2.0-18.8%) and new combinations 2.4% (25%-75%=0.7-9.1%).

The five most frequently implanted new stems were used in 9,049 THRs (49.8% of THRs using a new stem, Table S2). The five most frequently implanted new cups were used in 26,962THRs (75.1% of THRs using a new cup, Table S1). Uptake of the two most popular new cups was rapid (5,000 uses of Exeter X3 Rimfit 1,016 days, 5,000 uses of Trinity 1,651 days after first use, Figure 1) but was slower for new stems (2,000 uses of Polarstem Cementless 1,670 days, Figure 1). Conversely, a third of the new stems and cups (n=26/72 new stems, n=25/69 new cups) have been used in ≤10 THRs, and most of these have been used in ≤5 THRs (n=22 stems, n=20 cups).

Surgeon-level and patient-level factors associated with new implant components

Our complete case analysis included 431,955 out of a possible 618,393 THRs (69.8%) and 20,410 out of a possible 23,887 surgeon calendar-years (85.4%, Figure S2). We were missing patient-level data for BMI (n=186,308, 30.1%) and source of funding (n=1,514, 0.2%). The characteristics of the subset of patients with complete data are shown in Table S6. There were minor differences between people with complete data and those with incomplete data (Table S6). Compared with people with incomplete data, a smaller proportion of people with complete data were aged ≥80 years old (14.8% vs 16.4%), female (60.3% vs 61.6%) and had their operation funded through the NHS (86.9% vs 89.4%).

Characteristics of surgeons using new implant components

Multivariable associations between surgeon-level factors and their use of new components in a calendar-year were consistent between stems and cups (Table 1, unadjusted Table S7). Surgeons who treated more younger patients had 47% higher odds of using a new stem (OR=1.47, 95%CI 1.30-1.66, p<0.001) and 39% higher odds of using a new cup (OR=1.39, 95%CI 1.25-1.53, p<0.001) in a calendar-year. Those who performed more THRs/year had 6% higher odds of using new cups (OR=1.06, 95%CI 1.04-1.08, p<0.001) and 2% higher odds of using new stems (OR=1.02, 95%CI 1.00-1.05, p=0.03), although the confidence interval crossed the null. Private funding was associated with 23% increased odds of using new stems (OR=1.23, 95%CI 1.05-1.43, p=0.010) and weakly associated with 9% increased odds of using new cups (OR=1.09, 95%CI 0.96-1.23, p=0.187) with confidence intervals crossing the null value. Use of more stem-cup combinations was strongly associated with increased use of new components (ORs for '>10' vs. '≤3' combinations: 27.4 and 13.3 for stems and cups respectively, p values <0.001). Proportion of patients with ASA grades III-IV was weakly associated with 12% higher odds of

For peer review only - http://bmjopen.bmj.com/site/about/guidelines.xhtml

using new cups (OR=1.12, 95%CI 1.01-1.25, P=0.034) but not with using new stems (OR=1.01, 95%CI 0.89-1.16, p=0.843).

Characteristics of patients receiving new implant components

A higher proportion of recipients of new compared with established implant components were aged <55 years old (10.5% established vs. 21.3% new stems; 10.5% established vs. 14.8% new cups; Table 2), although the main recipients of all components were aged 55-80 years. Fifteen percent of recipients of established stems (15.0%) were ≥80 years old compared with 8.3% of recipients of new stems, but there was little difference in the proportion of older recipients of established (14.9%) and new (13.3%) cups. Across all components and component age, women were the main recipients of THRs. There was no difference in BMI between recipients of established and new stems or cups. A higher proportion of recipients of new components had ASA grade I (20.3% new vs. 14.3% established stems; 17.1% new vs. 14.3% established cups). A higher proportion of people with privately funded THRs had new components (stems: 19.6% new vs. 12.9% established; cups: 19.5% new vs. 12.7% established).

Multivariable mixed effects logistic regression models (Table 2, unadjusted Table S8) found that patients <55 years old, compared with those 55-80, had 83% and 31% higher odds of receiving a new rather than established stem (OR=1.83 95%CI 1.73-1.93, p<0.001) and cup (OR=1.31, 95%CI 1.25-1.37, p<0.001). Women had 13% lower odds than men of receiving a new stem (OR=0.87, 95%CI 0.84-0.90, p<0.001), but 6% higher odds of receiving a new cup (OR=1.06, 95%CI 1.03-1.09, p<0.001). There was weak evidence that people with higher BMI had 10% higher odds of receiving a new stem (OR for underweight/normal vs. Class II Obese=1.10, 95%CI 1.02-1.19, p=0.011) and weak evidence for the converse association between BMI and receiving a new cup (e.g. OR for underweight/normal vs. Class II Obese=0.94, 95%CI 0.89-1.00, p=0.042). Higher ASA grade was associated with 36% lower odds of receiving new stems (OR for ASA grades 'IV + V' versus 'I' = 0.64, 95%CI 0.46-0.90, p=0.010), but was not associated with receiving new cups (OR for ASA grades 'IV + V' versus 'I' = 1.02, 95%CI 0.82-1.26, p=0.881). Patients with private versus NHS funding had nine percent higher odds of receiving new cups (OR=1.09, 95%CI 1.04-1.14, p<0.001), but there was no association between source of funding and receiving new stems (OR=1.02, 95%CI 0.95-1.08, p=0.642).

Sensitivity analyses

Results of our first sensitivity analyses (excluding calendar-years for surgeons with <10 THRs) differed only minimally from our primary analyses (Table S9), indicating that our results were not biased by low-

For peer review only - http://bmjopen.bmj.com/site/about/guidelines.xhtml

volume surgeons. In our second sensitivity analyses ('consultant in-charge' as the clustering variable) associations between source of funding and receipt of new stem/cup were stronger, otherwise they differed only minimally differed from our primary analyses (Table S10 & Table S11). Our comparison of regression models without BMI as an exposure, with complete cases as defined previously (n=431,955) and complete cases defined without BMI (n=616,879) found only minor differences. This suggests that associations between the exposures and outcomes for the population missing BMI differ only slightly from the population with BMI.

Discussion

Sixty-eight new cups and 72 new stems were first used in THRs in the NJR for OA between 2008 and 2017. Most THRs used components introduced before 2008 but 12% used a new stem or cup. Uptake of some new implant components was very rapid. Conversely, uptake of a third of new components has been slow. Most surgeons used a maximum total of seven different cups or stems, of which one or two were new components. A small number of surgeons used a wide variety of different components, including new stems, cups and combinations.

Strengths of our study include the use of the NJR dataset, the largest arthroplasty register with comprehensive data capture (>95% in the period studied). This is the first to describe the variation in factors associated with uptake of new implant components by surgeons and receipt of new components by patients. Our study has several weaknesses. We classified a component as new based on the first record of a brand name in the NJR, but this does not exclude the possibility that a component was introduced earlier to other markets outside the UK. Furthermore, new components may constitute procedures not uploaded to the NJR (missing primary THRs estimated <5%). Also, some of these components may be minor modifications or a rebadged/renamed version of an existing component and some may also cover successive versions of a component. The correct operating surgeon may not be assigned to every operation. The extent to which this applies is unknown but may result in inaccurate estimates of surgeon-level associations, although our sensitivity analyses indicate that this is unlikely. The associations we have reported may be confounded by unmeasured factors (residual confounding) and in the absence of pre-existing literature on the uptake of new implants the findings from the regression models should be considered exploratory. We were missing BMI data for some people and elected not to use multiple imputation to account for these missing data, however our sensitivity

For peer review only - http://bmjopen.bmj.com/site/about/guidelines.xhtml

analyses suggest that people with BMI data did not differ substantially from those without BMI across our other measures. Finally, we did not have data on hospital-level factors or regional variation in suppliers in our analyses, which may be drivers of selection.[15]

Approximately 16 new implant components/year (stems and cups) were introduced in the NJR between 2008-2017. Comparisons with Australia (34 implant components/year 2003-2008) [16] and Finland (2-4 components/year 1980-2013) [17] suggest that this rate is not unusual, but that there is large variation internationally. The rapid uptake of some new components indicates that phased introduction, as recommended in the IDEAL Framework and others,[18] is unlikely to be happening. It is unclear whether 16 new implant components/year is of itself a good or bad thing. However, a healthcare system which supported a graduated introduction of new components, where the use of new components is restricted to specialised centres,[18] would provide a natural limit on the rate of introduction of new components until satisfactory and robust evidence is generated to support their more widespread use. Conversely, a third of new implant components have not yet accrued more than ten uses. Postmarket surveillance of THRs, due to their longevity, performs a safety monitoring role which cannot easily be replaced by pre-approval clinical data. Since the statistical methods are not applicable to components used in small numbers collaboration between international arthroplasty registries may allow more effective monitoring for low-volume components.

Over half of surgeons in our study used ≤5 different stems, cups, or combinations, similar to a median of two different implant brands reported by surgeons in the USA in 1997.[19] The volume of THRs performed by surgeons using new components was often low (median ≤3 THRs with new components versus median 22 THRs in total), but the proportion of their THRs using any new components varied from one percent (lower quartile) to 19% (upper quartile). Surgeons who use a wider range of prosthesis combinations in THRs may have higher revision rates [20] and early THRs performed after switching implants may have a higher revision risk (a.k.a. 'learning-curve').[21] While this suggests that surgeons should rely on a narrow range of implant components and rarely switch, a phased introduction of new implant designs, as is done in Sweden, may mitigate the learning-curve effect.[22] Since there are no contemporary comparisons of the range of implant components surgeons use and their relative volumes, it is unclear whether the between-surgeon variation we have reported may be associated with worse implant survival and warrants further research.

We found that newer components were being used in patients likely to be more active (i.e. younger and/or male patients). There has been increasing evidence that uncemented implants, particularly

For peer review only - http://bmjopen.bmj.com/site/about/guidelines.xhtml

stems, should not be used in older patients, but some uncertainty remains about their use in young patients (especially uncemented cups).[23–25] Since the majority of new cups and stems are uncemented, the decision to use these implant components in younger patients may increase the already high lifetime risk of revision surgery for these patients. Associations between BMI or ASA grade and receipt or use of new components were inconsistent between stems and cups and did not provide clear support for the use of new implant components in patients likely to be more active (i.e. lower BMI and ASA grades). It may be of interest to further investigate the implant component choices made for patients with higher BMI or ASA grades.

The most comparable previous work used NJR data to explore patient-level and hospital-level determinants that patients receive uncemented versus cemented implants.[15] Uncemented components were less likely to be used in women and older patients, and hospitals treating older patients were less likely to use them. Our results indicate that surgeons who treat a higher proportion of younger patients are more likely to use newer components. Our most marked finding, that surgeons who used a wide variety of stem-cup combinations (either established or new) were much more likely to try a new component, may be somewhat self-evident but suggests that there may be a subset of surgeons who change components more quickly than their peers. Whether this behaviour, alongside the previously discussed learning-curve, is related to outcomes of THRs is currently unclear.

Proposals for how new implant components should be introduced have been made previously, largely focussed on phased introduction through high-volume centres and surgeons, and reliance on registries for long-term monitoring. It seems unlikely that 16 new THR implant components/year, as we found in our study, could be sustained through such an approach. Alongside the potential benefits of phased introduction discussed elsewhere, this approach would probably reduce the number of implant components used only in very low numbers. Since these are not monitored in the same manner as higher volume components this would probably be a good thing for patients, providing implant components intended for use in specialist cases are not adversely affected.

Further research could build on the findings of this study in several ways. Extending our analysis of surgeon-level factors associated with uptake of new components to include factors associated with risk of revision after THR would be valuable to surgeons and patients. Specifically, the ‘learning curve’ associated with changing implants and the complex relationship between surgeon’s volume and outcomes. In addition, widening our study to cover hospital-level factors or regional variation in suppliers may highlight other drivers of selection.

For peer review only - http://bmjopen.bmj.com/site/about/guidelines.xhtml

Conclusions

A large number of new THR implant components have been introduced into use in the NJR since 2008. The majority of THRs performed since 2008 used components which have been in use for a long time, but a large number of surgeons have tried new components, with wide variation in how many types and how often they have been used. The impact of this variation on patient outcomes is currently unclear. New rather than established implant components are more likely to be used in patients who are younger and/or male, although whether this will reduce the high lifetime risk of revision for this population is unclear.

For peer review only

References

1 Dreinhöfer KE, Dieppe P, Stürmer T, *et al.* Indications for total hip replacement: Comparison of assessments of orthopaedic surgeons and referring physicians. *Annals of the Rheumatic Diseases* 2006;**65**:1346–50. doi:10.1136/ard.2005.047811

2 National Joint Registry for England, Wales and Northern Ireland. 14th Annual Report 2017. 2017.

3 National Institute for Health and Care Excellence. Total hip replacement and resurfacing arthroplasty for end-stage arthritis of the hip. NICE 2018.

4 Bayliss LE, Culliford D, Monk AP, *et al.* The effect of patient age at intervention on risk of implant revision after total replacement of the hip or knee: a population-based cohort study. *The Lancet* 2017;**389**:1424–30. doi:10.1016/S0140-6736(17)30059-4

5 Smith AJ, Dieppe P, Vernon K, *et al.* Failure rates of stemmed metal-on-metal hip replacements: Analysis of data from the National Joint Registry of England and Wales. *The Lancet* 2012;**379**:1199–204. doi:10.1016/S0140-6736(12)60353-5

6 de Steiger RN, Hang JR, Miller LN, *et al.* Five-Year Results of the ASR XL Acetabular System and the ASR Hip Resurfacing System. *The Journal of Bone and Joint Surgery-American Volume* 2011;**93**:2287–93. doi:10.2106/JBJS.J.01727

7 Reito A, Lehtovirta L, Lainiala O, *et al.* Lack of evidence—the anti-stepwise introduction of metal-on-metal hip replacements. *Acta Orthopaedica* 2017;**88**:478–83. doi:10.1080/17453674.2017.1353794

8 Kynaston-Pearson F, Ashmore AM, Malak TT, *et al.* Primary hip replacement prostheses and their evidence base: systematic review of literature. *BMJ* 2013;**347**:f6956. doi:10.1136/bmj.f6956

9 López-López JA, Humphriss RL, Beswick AD, *et al.* Choice of implant combinations in total hip replacement: systematic review and network meta-analysis. *BMJ (Clinical research ed)* 2017;**359**:j4651. doi:10.1136/bmj.j4651

For peer review only - http://bmjopen.bmj.com/site/about/guidelines.xhtml

10 McCulloch P, Altman DG, Campbell WB, *et al.* No surgical innovation without evaluation: the IDEAL recommendations. *The Lancet* 2009;**374**:1105–12. doi:10.1016/S0140-6736(09)61116-8

11 Birkmeyer JD, Reames BN, McCulloch P, *et al.* Understanding of regional variation in the use of surgery. *The Lancet* 2013;**382**:1121–9. doi:10.1016/S0140-6736(13)61215-5

12 National Joint Registry for England W and NI. Prostheses used in hip, knee, ankle, elbow and shoulder replacement procedures 2016. 2017.

13 National Joint Registry for England, Wales and Northern Ireland. Data Completeness and quality. 2018.http://www.njrreports.org.uk/Data-Completeness-and-quality (accessed 7 Aug 2019).

14 National Joint Registry for England. 15th Annual Report 2018. 2018. http://www.njrreports.org.uk/Portals/0/PDFdownloads/NJR%2015th%20Annual%20Report%202018.pdf (accessed 30 Apr 2019).

15 Davies C. *An analysis of choice: a case study on hip prostheses*. 2011.

16 Anand R, Graves SE, de Steiger RN, *et al.* What Is the Benefit of Introducing New Hip and Knee Prostheses? *The Journal of Bone and Joint Surgery-American Volume* 2011;**93**:51–4. doi:10.2106/JBJS.K.00867

17 Peltola M. *Impact of technological change on quality of care: Studies on total hip and knee replacement*. 2016.

18 Zywiel MG, Johnson AJ, Mont MA. Graduated Introduction of Orthopaedic Implants. *The Journal of Bone & Joint Surgery* 2012;**94**:e158. doi:10.2106/JBJS.K.01675

19 Sharkey PF, Sethuraman V, Hozack WJ, *et al.* Factors influencing choice of implants in total hip arthroplasty and total knee arthroplasty: Perspectives of surgeons and patients. *Journal of Arthroplasty* 1999;**14**:281–7. doi:10.1016/S0883-5403(99)90052-9

20 Australian Orthopaedic Association. Hip, Knee & Shoulder Arthroplasty: Annual Report 2017. Australian Orthopaedic Association 2017. https://aoanjrr.sahmri.com/documents/10180/397736/Hip%2C%20Knee%20%26%20Shoulder%20 Arthroplasty

21 Peltola M, Malmivaara A, Paavola M. Hip prosthesis introduction and early revision risk. *Acta Orthopaedica* 2013;**84**:25–31. doi:10.3109/17453674.2013.771299

22 Mohaddes M, Björk M, Nemes S, *et al.* No increased risk of early revision during the implementation phase of new cup designs: Analysis of 52,903 hip arthroplasties reported to the Swedish Hip Arthroplasty Register. *Acta Orthopaedica* 2016;**87**:31–6. doi:10.1080/17453674.2016.1181818

23 Mäkelä KT, Matilainen M, Pulkkinen P, *et al.* Failure rate of cemented and uncemented total hip replacements: Register study of combined Nordic database of four nations. *BMJ (Online)* 2014;**348**:1–10. doi:10.1136/bmj.f7592

For peer review only - http://bmjopen.bmj.com/site/about/guidelines.xhtml

24 Stea S, Comfort T, Sedrakyan A, *et al.* Multinational comprehensive evaluation of the fixation method used in hip replacement: Interaction with age in context. *Journal of Bone and Joint Surgery - American Volume* 2014;**96**:42–51. doi:10.2106/JBJS.N.00463

25 Hailer NP, Garellick G, Kärrholm J. Uncemented and cemented primary total hip arthroplasty in the Swedish Hip Arthroplasty Register: Evaluation of 170,413 operations. *Acta Orthopaedica* 2010;**81**:34–41. doi:10.3109/17453671003685400

For peer review only

For peer review only - http://bmjopen.bmj.com/site/about/guidelines.xhtml

Table 1 Results from multivariable logistic regression models showing the association between surgeon-level factors and use of new stems and cups

	Stems					Cups				
**Exposure**	**Established** (n=18,404)[3]	**New** (n=2,006)[3]	**OR**[1]	**(95% CI)**	**p**[1]	**Established** (n=17,167)[3]	**New** (n=3,243)[3]	**OR**[1]	**(95% CI)**	**p**[1]
**Proportion of THRs performed on patients <55 years old**										
<10% (ref.)	13,088 (71.1%)	940 (46.9%)	1	-	-	12,346 (71.9%)	1,682 (51.9%)	1	-	-
≥10%	5,316 (28.9%)	1,066 (53.1%)	1.47	1.30 – 1.66	<0.001	4,821 (28.1%)	1,561 (48.1%)	1.39	1.25 – 1.53	<0.001
**Number of THRs performed in calendar year**[2] (per 10 additional cases)	8 (2, 24)	32 (12, 61)	1.02	0.99 – 1.04	0.206	7 (2, 22)	28 (10, 56)	1.06	1.04 – 1.08	<0.001
**Proportion of THRs funded privately**										
100% NHS funded (ref.)	12,922 (70.2%)	966 (48.2%)	1	-	-	12,159 (70.8%)	1,729 (53.3%)	1	-	-
Some or all funded privately	5,482 (29.8%)	1,040 (51.8%)	1.23	1.05 – 1.43	0.010	5,008 (29.2%)	1,514 (46.7%)	1.09	0.96 – 1.23	0.187
**Number of stem-cup combinations used in calendar year**										
≤3 (ref.)	14,259 (77.5%)	589 (29.4%)	1	-	-	13,599 (79.2%)	1,249 (38.5%)	1	-	-
4-6	3,394 (18.4%)	822 (41.0%)	4.91	4.25 – 5.67	<0.001	2,937 (17.1%)	1,279 (39.4%)	3.77	3.36 – 4.23	<0.001
7-10	675 (3.7%)	468 (23.3%)	12.5	10.1 – 15.4	<0.001	568 (3.3%)	575 (17.7%)	7.21	6.01 – 8.67	<0.001

For peer review only - http://bmjopen.bmj.com/site/about/guidelines.xhtml

>10	76 (0.4%)	127 (6.3%)	27.4	17.9 – 41.7	<0.001	63 (0.4%)	140 (4.3%)	13.3	9.20 – 19.2	<0.001
**Proportion of THRs performed on patients with ASA grade III-V**										
<25% (ref.)	13,244 (72.0%)	1,554 (77.5%)	1	-	-	12,362 (72.0%)	2,436 (75.1%)	1	-	-
≥25%	5,160 (28.0%)	452 (22.5%)	1.01	0.89 – 1.16	0.843	4,805 (28.0%)	807 (24.9%)	1.12	1.01 – 1.25	0.034

1 – odds ratios, 95% confidence intervals and p-values are from logistic regression models adjusted for all exposure variables, 2 - median (lower to upper quartile), 3 - proportions displayed are based on surgeon-calendar years

For peer review only

For peer review only - http://bmjopen.bmj.com/site/about/guidelines.xhtml

Table 2 Results from multivariable mixed-effects regression models (patients nested within surgeons) of age, gender, categorised BMI, ASA grade and source of funding on stem age and cup age, with category proportions

	**Stems**					**Cups**				
	**Established (n=418,831)**	**New (n=13,124)**	**OR[1]**	**(95% CI)**	**p**	**Established (n=406,072)**	**New (n=25,883)**	**OR[1]**	**(95% CI)**	**p**
**Age**										
<55 years old	43,780 (10.5%)	2,793 (21.3%)	1.83	1.73 – 1.93	<0.001	42,752 (10.5%)	3,821 (14.8%)	1.31	1.25 – 1.37	<0.001
55 to 80 (ref.)	312,205 (74.5%)	9,246 (70.5%)	1	-	-	302,823 (74.6%)	18,628 (72.0%)	1	-	-
≥ 80 years old	62,846 (15.0%)	1,085 (8.3%)	0.60	0.56 – 0.64	<0.001	60,497 (14.9%)	3,434 (13.3%)	0.91	0.87 – 0.95	<0.001
**Gender**										
Male (ref.)	165,607 (39.5%)	5,768 (44.0%)	1	-	-	161,248 (39.7%)	10,127 (39.1%)	1	-	-
Female	253,224 (60.5%)	7,356 (56.0%)	0.87	0.84 – 0.90	<0.001	244,824 (60.3%)	15,756 (60.9%)	1.06	1.03 – 1.09	<0.001
**BMI**										
Underweight and normal (ref.)	95,306 (22.8%)	2,911 (22.2%)	1	-	-	91,863 (22.6%)	6,354 (24.5%)	1	-	-
Overweight	165,849 (39.6%)	5,138 (39.1%)	1.02	0.97 – 1.08	0.373	160,834 (39.6%)	10,153 (39.2%)	0.95	0.91 – 0.99	0.007
Class I Obese	105,670 (25.2%)	3,391 (25.8%)	1.06	1.00 – 1.12	0.067	102,781 (25.3%)	6,280 (24.3%)	0.93	0.90 – 0.97	0.001
Class II Obese	38,995 (9.3%)	1,276 (9.7%)	1.10	1.02 – 1.19	0.011	37,977 (9.4%)	2,294 (8.9%)	0.94	0.89 – 1.00	0.042
Class III Obese	13,011 (3.1%)	408 (3.1%)	0.99	0.87 – 1.11	0.808	12,617 (3.1%)	802 (3.1%)	0.94	0.86 – 1.02	0.135
**ASA grade**										
I (ref.)	60,022 (14.3%)	2,661 (20.3%)	1	-	-	58,265 (14.3%)	4,418 (17.1%)	1	-	-
II	293,142 (70.0%)	8,940 (68.1%)	0.81	0.77 – 0.86	<0.001	284,437 (70.0%)	17,645 (68.2%)	0.98	0.95 – 1.03	0.461

For peer review only

III	63,904 (15.3%)	1,482 (11.3%)	0.66	0.61 – 0.72	<0.001	61,681 (15.2%)	3,705 (14.3%)	1.00	0.94 – 1.05	0.935
IV + V	1,763 (0.4%)	41 (0.3%)	0.64	0.46 – 0.90	0.010	1,689 (0.4%)	115 (0.4%)	1.02	0.82 – 1.26	0.881
**Source of funding**										
NHS	364,928 (87.1%)	10,553 (80.4%)	1	-	-	354,642 (87.3%)	20,839 (80.5%)	1	-	-
Private	53,903 (12.9%)	2,571 (19.6%)	1.02	0.95 – 1.08	0.642	51,430 (12.7%)	5,044 (19.5%)	1.09	1.04 – 1.14	<0.001

1 – odds ratios, 95% confidence intervals and p-values are from mixed-effects logistic regression models adjusted for all exposure variables

For peer review only

Figure 1 Cumulative total use of the top 5 new stems and cups/shells by days since they were introduced

For peer review only

For peer review only - http://bmjopen.bmj.com/site/about/guidelines.xhtml

For peer review only

For peer review only - http://bmjopen.bmj.com/site/about/guidelines.xhtml

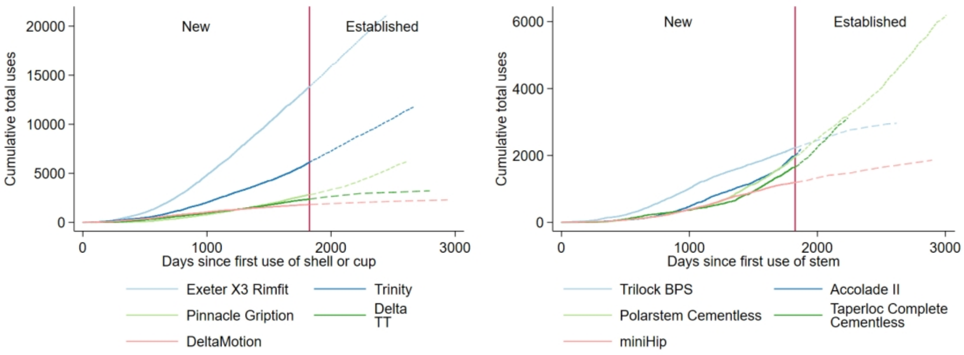

Figure 1 Cumulative total use of the top 5 new stems and cups/shells by days since they were introduced

674x269mm (72 x 72 DPI)

Supplementary material

Table S1 Uptake of new cups first used between January 1st 2008 and 26th February 2017

Cup/shell brand	UC [1]	Patients	Percent	Surgeons	Month first used
**Exeter X3 Rimfit**		**13,821**	**38.5%**	**781**	**Jun 2010**
**Trinity**	✓	**6,133**	**17.1%**	**197**	**Nov 2009**
**Pinnacle Gription**	✓	**2,817**	**7.9%**	**375**	**Dec 2009**
**Delta TT**	✓	**2,368**	**6.6%**	**145**	**Jun 2009**
**DeltaMotion**	✓	**1,823**	**5.1%**	**152**	**Feb 2009**
Versafit CC Trio	✓	1,442	4.0%	47	Mar 2011
RM Pressfit Vitamys	✓	731	2.0%	33	Aug 2011
G7 Cementless Acetabular Component	✓	621	1.7%	36	Aug 2014
AEON Cemented Acetabular Cup		568	1.6%	43	Sep 2011
Exceed ABT Cemented		556	1.5%	57	Jun 2011
Plasmafit Cementless Cup	✓	546	1.5%	50	Nov 2012
Duracel		465	1.3%	45	Mar 2013
Allofit IT	✓	367	1.0%	19	Jan 2010
ADES Cemented		342	1.0%	72	Feb 2014
XLFit Acetabular Cup	✓	293	0.8%	56	Apr 2015
Regenerex Ringloc+	✓	220	0.6%	55	Feb 2009
April - Polyethylene	✓	214	0.6%	33	Jan 2012
ADES	✓	205	0.6%	39	May 2014
Delta PF	✓	198	0.6%	9	Mar 2011
MIHR Cup	✓	197	0.5%	12	Mar 2008
RM Pressfit	✓	184	0.5%	24	May 2008
Tribofit	✓	174	0.5%	9	Jul 2010
seleXys TH+	✓	174	0.5%	13	Nov 2008
OptiCup CEP		147	0.4%	18	Nov 2014
Delta One TT	✓	129	0.4%	61	Jun 2010
Gyros	✓	129	0.4%	28	Jan 2010
Restoration ADM Cup	✓	127	0.4%	31	May 2011
EcoFit Cementless Cup	✓	102	0.3%	5	Feb 2013
Novation	✓	93	0.3%	10	Nov 2009
Allofit-S IT	✓	91	0.3%	21	Aug 2010
M2A Magnum	✓	79	0.2%	30	Feb 2008
Freedom		75	0.2%	17	May 2008
Captiv DM	✓	68	0.2%	8	Aug 2011
Trident Constrained Cup		65	0.2%	30	Jan 2008

For peer review only

seleXys DS Cementless	✓	56	0.2%	16	Mar 2014
MMC Resurfacing	✓	36	0.1%	10	Aug 2009
ASR 300 Cup	✓	36	0.1%	1	Jan 2009
MPACT	✓	25	0.1%	8	Dec 2011
Restoration Gap2		23	0.1%	14	Mar 2008
Fixa Ti-Por	✓	20	0.1%	4	Apr 2014
seleXys DS Cemented		20	0.1%	12	Feb 2014
Fixa Duplex	✓	17	0.0%	1	Mar 2016
Cormet Prime	✓	12	0.0%	5	Jan 2010
Delta Revision TT	✓	9	0.0%	6	Nov 2010
Equateur	✓	9	0.0%	5	Jul 2008
U-Motion II	✓	8	0.0%	4	Apr 2016
A Class		7	0.0%	3	Feb 2009
Zimmer Cemented Cup		6	0.0%	4	May 2013
Regenerex Revision	✓	4	0.0%	3	Jan 2009
Capitole C		3	0.0%	3	Jan 2013
Sirius Cementless Cup	✓	3	0.0%	2	Aug 2011
Horizon	✓	3	0.0%	2	Jul 2008
2M Dual Mobility	✓	3	0.0%	2	Nov 2012
Par-5	✓	3	0.0%	3	Jan 2008
Solution Cemented Cup		2	0.0%	1	Dec 2015
J-Loc	✓	2	0.0%	2	Mar 2013
XPE Cup		2	0.0%	1	Jun 2016
Evidence		2	0.0%	1	Oct 2014
FIXA Duplex Cemented		1	0.0%	1	Jan 2017
Endurance Cemented Cup		1	0.0%	1	Oct 2016
Mitre Cup		1	0.0%	1	Nov 2013
Capitole T	✓	1	0.0%	1	Nov 2014
Polymax	✓	1	0.0%	1	Oct 2016
Ringloc	✓	1	0.0%	1	Jan 2011
Charnley KS		1	0.0%	1	Jul 2011
Arden		1	0.0%	1	Feb 2008
Versacem		1	0.0%	1	Oct 2009
Versafit DM	✓	1	0.0%	1	May 2008
Total		35,885			

1 – Uncemented fixation, Rows in **bold** = five most commonly used new cups

For peer review only

Table S2 Uptake of new stems first used between January 1st 2008 and 26th February 2017

Stem brand	UC [1]	Patients	Percent	Surgeons	Month first used
**Trilock BPS**	✓	**2,232**	**12.3%**	**121**	**Dec 2009**
**Accolade II**	✓	**1,997**	**11.0%**	**165**	**Jan 2012**
**Polarstem Cementless**	✓	**1,969**	**10.8%**	**93**	**Dec 2008**
**Taperloc Complete Cementless Stem**	✓	**1,658**	**9.1%**	**104**	**Jan 2011**
**miniHip**	✓	**1,193**	**6.6%**	**79**	**Mar 2009**
Metafix Stem	✓	1,171	6.4%	93	Feb 2008
AMIStem-H	✓	1,003	5.5%	32	Aug 2009
Exeter No.1 125mm stem Line Extension		836	4.6%	211	Aug 2014
TriFit TS hip stem	✓	684	3.8%	44	Sep 2012
Aeon Cemented Stem		673	3.7%	50	Sep 2011
SPS Evolution	✓	654	3.6%	48	Jan 2012
C-Stem AMT Line Extension		428	2.4%	127	Jul 2013
H-Max S Monoblock Stem	✓	401	2.2%	34	May 2010
H-Max M Modular Stem	✓	316	1.7%	20	Mar 2010
Finsbury Type C	✓	302	1.7%	39	Aug 2008
EcoFit Cementless Stem	✓	240	1.3%	11	Sep 2010
Silent	✓	199	1.1%	17	Feb 2008
Metha Monoblock Stem	✓	195	1.1%	25	Aug 2011
Corail Cemented		170	0.9%	32	Apr 2009
OptiStem		165	0.9%	22	Nov 2014
Trilliance		156	0.9%	10	Jul 2011
Sirius stem		138	0.8%	10	Apr 2014
Profemur L Classic	✓	132	0.7%	17	Mar 2014
Profemur TL	✓	120	0.7%	23	Jan 2008
AMIStem-C		110	0.6%	3	Jul 2012
Master SL	✓	102	0.6%	8	Jul 2013
Corail Revision Stem	✓	92	0.5%	69	Jul 2010
Novation Element Stem	✓	90	0.5%	9	Nov 2009
CBC Evolution	✓	83	0.5%	8	Jan 2013
Nanos	✓	78	0.4%	5	Dec 2011
Amoda	✓	67	0.4%	1	Apr 2010
Harmony Modular	✓	65	0.4%	6	Mar 2010
ABG II Cementless Stem	✓	52	0.3%	9	Apr 2009
SL	✓	51	0.3%	5	Sep 2009
XActa		47	0.3%	5	Jan 2014
Avenir Muller Cementless	✓	33	0.2%	6	Jun 2016
Harmony Cemented		25	0.1%	8	Feb 2014

For peer review only

miniMax	✓	24	0.1%	2	Apr 2011
SMS	✓	22	0.1%	2	Jul 2015
FTS	✓	20	0.1%	5	Feb 2009
Profemur TL Classic	✓	19	0.1%	5	Jan 2016
SMF	✓	17	0.1%	1	Oct 2011
Profemur Preserve	✓	12	0.1%	5	Feb 2012
AMIStem HP	✓	12	0.1%	1	Dec 2015
METS Cemented		12	0.1%	10	Dec 2012
GMRS		11	0.1%	9	Aug 2012
Harmony Cementless	✓	10	0.1%	4	Apr 2011
UCP Stem		10	0.1%	5	Apr 2016
Echelon Cemented Stem		8	0.0%	6	Mar 2008
Exception Cementless	✓	6	0.0%	3	Feb 2010
METS Cementless	✓	5	0.0%	5	Feb 2013
Novation Stem	✓	5	0.0%	2	Mar 2014
G2 Cementless Stem	✓	5	0.0%	5	Dec 2013
Securus	✓	4	0.0%	4	Dec 2009
Profemur Gladiator	✓	4	0.0%	3	Mar 2010
Arcad Cementless	✓	4	0.0%	4	Sep 2010
Euros Cementless	✓	3	0.0%	2	Aug 2011
Atlantis	✓	3	0.0%	3	Dec 2011
Quadra-C		3	0.0%	2	Oct 2009
Restoration Cemented Stem		1	0.0%	1	Feb 2014
Wagner Revision Stem	✓	1	0.0%	1	Apr 2016
Initiale Cemented Stem		1	0.0%	1	Jul 2008
Integrale	✓	1	0.0%	1	Jun 2009
optimys	✓	1	0.0%	1	Feb 2017
Prodigy	✓	1	0.0%	1	Jul 2010
CDH Stem	✓	1	0.0%	1	Nov 2012
Friendly		1	0.0%	1	Jul 2012
Regulus Cemented Stem		1	0.0%	1	Oct 2016
Arcad Cemented		1	0.0%	1	Feb 2009
Endurance Cemented Stem		1	0.0%	1	Sep 2013
Furlong HAC Hemiarthroplasty	✓	1	0.0%	1	Oct 2010
C2 Stem	✓	1	0.0%	1	Feb 2015
**Total**		**18,159**			

1 – Uncemented fixation, Rows in **bold** = five most commonly used new stems

For peer review only

Table S3 Number of different post-2008 shells/cups used by surgeons

Number of new cups used	Number of surgeons	Percent	Cumulative percent
1	1,113	65.5%	65.5%
2	351	20.7%	86.2%
3	138	8.1%	94.3%
4	61	3.6%	97.9%
5	18	1.1%	98.9%
6	8	0.5%	99.4%
7	9	0.5%	99.9%
10	1	0.1%	100.0%
**Total**	1,699		

For peer review only

Table S4 Number of different post-2008 stems used by surgeons

Number of new stems used	Number of surgeons	Percent	Cumulative percent
1	771	69.4%	69.4%
2	210	18.9%	88.3%
3	77	6.9%	95.2%
4	33	3.0%	98.2%
5	9	0.8%	99.0%
6	8	0.7%	99.7%
7	1	0.1%	99.8%
8	2	0.2%	100.0%
**Total**	1,111		

For peer review only

Table S5 The number of unique new stem-cup combinations used simultaneously by surgeons

Stem-cup combinations	Number of surgeons	Percent	Cumulative percent
1	311	71.8%	71.8%
2	78	18.0%	89.8%
3	22	5.1%	94.9%
4	10	2.3%	97.2%
5	5	1.2%	98.4%
6	2	0.5%	98.9%
7	1	0.2%	99.1%
8	2	0.5%	99.5%
9	2	0.5%	100.0%
**Total**	433	100%	

For peer review only

Table S6 A comparison of people with complete data and those missing some data

	Incomplete (n=186,438)	Complete (n=431,995)	P*
**Age**			
<55 years old	19,561 (10.5%)	46,573 (10.8%)	<0.001
55 to 80	136,370 (73.1%)	321,451 (74.4%)	
≥ 80 years old	30,507 (16.4%)	63,931 (14.8%)	
**Gender**			
Male	71,676 (38.4%)	171,375 (39.7%)	<0.001
Female	114,762 (61.6%)	260,580 (60.3%)	
**BMI**			
Underweight and normal	31 (23.8%)	98,217 (22.7%)	0.097
Overweight	43 (33.1%)	170,987 (39.6%)	
Class I Obese	33 (25.4%)	109,061 (25.2%)	
Class II Obese	14 (10.8%)	40,271 (9.3%)	
Class III Obese	9 (6.9%)	13,419 (3.1%)	
**ASA grade**			
I	24,893 (13.4%)	62,683 (14.5%)	<0.001
II	130,223 (69.8%)	302,082 (69.9%)	
III	30,180 (16.2%)	65,386 (15.1%)	
IV + V	1,142 (0.6%)	1,804 (0.4%)	
**Source of funding**			
NHS	165,394 (89.4%)	375,481 (86.9%)	<0.001
Private	19,530 (10.6%)	5,6474 (13.1%)	

*- P-values from chi square tests

For peer review only

Table S7 Results from unadjusted logistic regression models showing the association between surgeon-level factors and use of new versus old stems and cups

	Stems			Cups		
**Exposure**	**OR[1]**	**(95% CI)**	**p**	**OR[1]**	**(95% CI)**	**p**
**Proportion of THRs performed on patients <55 years old**						
<10% (ref.)	1	-	-	1	-	-
≥10%	2.79	2.49 – 3.13	<0.001	2.38	2.16 – 2.62	<0.001
**Number of THRs performed in calendar year[2]** (per 10 additional cases)	1.19	1.16 – 1.22	<0.001	1.20	1.18 – 1.23	<0.001
**Proportion of THRs funded privately**						
100% NHS funded (ref.)	1	-	-	1	-	-
Some or all funded privately	2.54	2.21 – 2.91	<0.001	2.13	1.90 – 2.37	<0.001
**Number of stem-cup combinations used in calendar year**						
≤3 (ref.)	1	-	-	1	-	-
4-6	5.86	5.16 – 6.66	<0.001	4.74	4.27 – 5.26	<0.001
7-10	16.8	14.1 – 20.0	<0.001	11.0	9.39 – 12.9	<0.001
>10	40.5	27.2 – 60.1	<0.001	21.2	17.1 – 34.2	<0.001
**Proportion of THRs performed on patients with ASA grade III-V**						
<25% (ref.)	1	-	-	1	-	-
≥25%	0.75	0.66 – 0.85	<0.001	0.85	0.77 – 0.95	0.003

1 – odds ratios, 95% confidence intervals and p-values are from unadjusted logistic regression models

For peer review only

Table S8 Results from unadjusted mixed-effects regression models (patients nested within surgeons) of age, gender, categorised BMI, ASA grade, and source of funding on stem and cup age

	Stems			Cups		
	**OR[1]**	**(95% CI)**	**p**	**OR[1]**	**(95% CI)**	**p**
**Age** (years)						
<55 years old	1.95	1.85 – 2.05	<0.001	1.31	1.25 – 1.36	<0.001
55 to 80 (ref.)	1	-	-	1	-	-
≥ 80 years old	0.56	0.52 – 0.60	<0.001	0.93	0.89 – 0.97	<0.001
**Gender**						
Male (ref.)	1	-	-	1	-	-
Female	0.83	0.80 – 0.86	<0.001	1.05	1.02 – 1.08	0.001
**BMI**						
Underweight and normal (ref.)	1	-	-	1	-	-
Overweight	1.06	1.01 – 1.12	0.017	0.94	0.91 – 0.98	0.001
Class I Obese	1.12	1.06 – 1.18	<0.001	0.93	0.90 – 0.97	0.001
Class II Obese	1.18	1.09 – 1.27	<0.001	0.96	0.90 – 1.01	0.123
Class III Obese	1.04	0.93 – 1.17	0.513	0.97	0.89 – 1.05	0.456
**ASA grade**						
I (ref.)	1	-	-	1	-	-
II	0.69	0.65 – 0.72	<0.001	0.92	0.88 – 0.96	<0.001
III	0.51	0.48 – 0.55	<0.001	0.90	0.86 – 0.95	<0.001
IV + V	0.47	0.33 – 0.65	<0.001	0.91	0.74 – 1.13	0.390
**Source of funding**						
NHS	1	-	-	1	-	-
Private	1.01	0.95 – 1.07	0.777	1.08	1.03 – 1.13	0.001

1 – odds ratios, 95% confidence intervals and p-values are from unadjusted mixed-effects logistic regression models

For peer review only

Table S9 Sensitivity analysis 1: Results from multivariable logistic regression models showing the association between surgeon-level factors and use of new versus old stems and cups, excluding surgeon calendar-years with <10 THRs

	Stems			Cups		
**Exposure**	**OR[1]**	**(95% CI)**	**p**	**OR[1]**	**(95% CI)**	**p**
**Proportion of THRs performed on patients <55 years old**						
<10% (ref.)	1	-	-	1	-	-
≥10%	1.30	1.13 – 1.49	<0.001	1.36	1.21 – 1.54	<0.001
**Number of THRs performed in calendar year[2]** (per 10 additional cases)	1.01	0.99 – 1.04	0.359	1.04	1.01 – 1.06	0.001
**Proportion of THRs funded privately**						
100% NHS funded (ref.)	1	-	-	1	-	-
Some or all funded privately	1.24	1.05 – 1.47	0.012	1.04	0.91 – 1.19	0.546
**Number of stem-cup combinations used in calendar year**						
≤3 (ref.)	1	-	-	1	-	-
4-6	4.13	3.44 – 4.96	<0.001	3.22	2.82 – 3.69	<0.001
7-10	10.8	8.62 – 13.6	<0.001	6.23	5.15 – 7.53	<0.001
>10	24.3	15.8 – 37.4	<0.001	12.1	8.37 – 17.5	<0.001
**Proportion of THRs performed on patients with ASA grade III-V**						
<25% (ref.)	1	-	-	1	-	-
≥25%	1.11	0.94 – 1.31	0.214	1.21	1.05 – 1.38	0.007

1 – odds ratios, 95% confidence intervals and p-values are from logistic regression models adjusted for all exposure variables

For peer review only

Table S10Sensitivity analysis 2a: Results from multivariable logistic regression models showing the association between surgeon-level factors (consultant in-charge) and use of new versus old stems and cups

	Stems			Cups		
**Exposure**	**OR[1]**	**(95% CI)**	**p**	**OR[1]**	**(95% CI)**	**p**
**Proportion of THRs performed on patients <55 years old**						
<10% (ref.)	1	-	-	1	-	-
≥10%	1.52	1.33 – 1.74	<0.001	1.46	1.30 – 1.64	<0.001
**Number of THRs performed in calendar year[2]** (per 10 additional cases)	1.02	1.00 – 1.04	0.039	1.05	1.04 – 1.07	<0.001
**Proportion of THRs funded privately**						
100% NHS funded (ref.)	1	-	-	1	-	-
Some or all funded privately	1.27	1.08 – 1.50	0.004	1.15	1.01 – 1.31	0.042
**Number of stem-cup combinations used in calendar year**						
≤3 (ref.)	1	-	-	1	-	-
4-6	4.72	3.98 – 5.60	<0.001	3.47	3.04 – 3.97	<0.001
7-10	11.2	8.87 – 14.1	<0.001	6.16	5.06 – 7.49	<0.001
>10	26.7	17.9 – 39.9	<0.001	11.3	7.91 – 16.1	<0.001
**Proportion of THRs performed on patients with ASA grade III-V**						
<25% (ref.)	1	-	-	1	-	-
≥25%	1.15	0.98 – 1.35	0.077	1.13	0.99 – 1.29	0.064

1 – odds ratios, 95% confidence intervals and p-values are from logistic regression models adjusted for all exposure variables

For peer review only

Table S11 Sensitivity analysis 2b: Results from multivariable mixed-effects regression models (patients nested within 'consultant in-charge') of age, gender, categorised BMI, ASA grade, and source of funding on stem and cup age

	**Stems**			**Cups**		
	**OR[1]**	**(95% CI)**	**p**	**OR[1]**	**(95% CI)**	**p**
**Age** (years)						
<55 years old	1.89	1.79 – 1.99	<0.001	1.32	1.26 – 1.37	<0.001
55 to 80 (ref.)	1	-	-	1	-	-
≥ 80 years old	0.59	0.55 – 0.63	<0.001	0.92	0.88 – 0.96	<0.001
**Gender**						
Male (ref.)	1	-	-	1	-	-
Female	0.87	0.84 – 0.91	<0.001	1.05	1.02 – 1.09	<0.001
**BMI**						
Underweight and normal (ref.)	1	-	-	1	-	-
Overweight	1.01	0.96 – 1.07	0.663	0.95	0.91 – 0.98	0.005
Class I Obese	1.05	0.99 – 1.11	0.123	0.93	0.89 – 0.97	<0.001
Class II Obese	1.09	1.01 – 1.17	0.031	0.94	0.89 – 1.00	0.039
Class III Obese	0.98	0.87 – 1.10	0.737	0.95	0.87 – 1.04	0.264
**ASA grade**						
I (ref.)	1	-	-	1	-	-
II	0.80	0.76 – 0.84	<0.001	0.97	0.93 – 1.01	0.168
III	0.64	0.60 – 0.69	<0.001	0.97	0.92 – 1.03	0.338
IV + V	0.62	0.44 – 0.87	0.006	1.00	0.81 – 1.23	0.981
**Source of funding**						
NHS	1	-	-	1	-	-
Private	1.14	1.07 – 1.21	<0.001	1.13	1.08 – 1.18	<0.001

1 – odds ratios, 95% confidence intervals and p-values are from mixed-effects logistic regression models adjusted for all exposure variables

For peer review only

Table S11 Sensitivity analysis 3: A comparison of results from multivariable mixed-effects regression models (patients nested within 'lead surgeon', excluding BMI) using a) patients with complete data for all exposures and BMI and, b) patients with complete data for all exposures excluding BMI

	**Stems**						**Cups**					
	**a) Complete cases only[1] (n = 431,955)**			**b) All cases[2] (n = 616,879)**			**a) Complete cases only[1] (n = 431,955)**			**b) All cases[2] (n = 616,879)**		
	**OR[3]**	**(95% CI)**	**p**	**OR[3]**	**(95% CI)**	**p**	**OR[3]**	**(95% CI)**	**p**	**OR[3]**	**(95% CI)**	**p**
**Age** (years)												
<55 years old	1.83	1.74 – 1.93	<0.001	1.81	1.73 – 1.90	<0.001	1.31	1.25 – 1.37	<0.001	1.37	1.32 – 1.42	<0.001
55 to 80 (ref.)	1	-	-	1	-	-	1	-	-	1	-	-
≥ 80 years old	0.59	0.55 – 0.63	<0.001	0.61	0.61 – 0.57	<0.001	0.92	0.88 – 0.96	<0.001	0.95	0.92 – 0.99	0.008
**Gender**												
Male (ref.)	1	-	-	1	-	-	1	-	-	1	-	-
Female	0.86	0.83 – 0.90	<0.001	0.84	0.82 – 0.87	<0.001	1.07	1.04 – 1.10	<0.001	1.04	1.02 – 1.07	0.001
**ASA grade**												
I (ref.)	1	-	-	1	-	-	1	-	-	1	-	-
II	0.82	0.78 – 0.87	<0.001	0.82	0.78 – 0.86	<0.001	0.98	0.94 – 1.02	0.264	0.97	0.94 – 1.01	0.100
III	0.67	0.62 – 0.72	<0.001	0.66	0.62 – 0.70	<0.001	0.99	0.94 – 1.04	0.641	1.01	0.97 – 1.06	0.591
IV + V	0.65	0.46 – 0.91	0.011	0.67	0.52 – 0.86	0.002	1.01	0.82 – 1.25	0.927	1.12	0.95 – 1.32	0.175
**Source of funding**												
NHS (ref.)	1	-	-	1	-	-	1	-	-	1	-	-
Private	1.01	0.95 – 1.08	0.688	1.02	0.97 – 1.08	0.403	1.09	1.04 – 1.14	<0.001	1.07	1.03 – 1.11	0.001

1 – The study sample for 'Complete cases only' was defined as those cases with complete data for all exposure variables (age, gender, ASA grade and source of funding) and BMI

2 - The study sample for 'All cases' was defined as those cases with complete data for all exposure variables (age, gender, ASA grade and source of funding)

3 – odds ratios, 95% confidence intervals and p-values are from mixed-effects logistic regression models adjusted for all exposure variables

For peer review only

Figure S1 The cumulative introduction of new brands of cup and stem components for THRs, between January 1st 2008 and 26th February 2017

For peer review only

Figure S2 STROBE Flow diagram

For peer review only

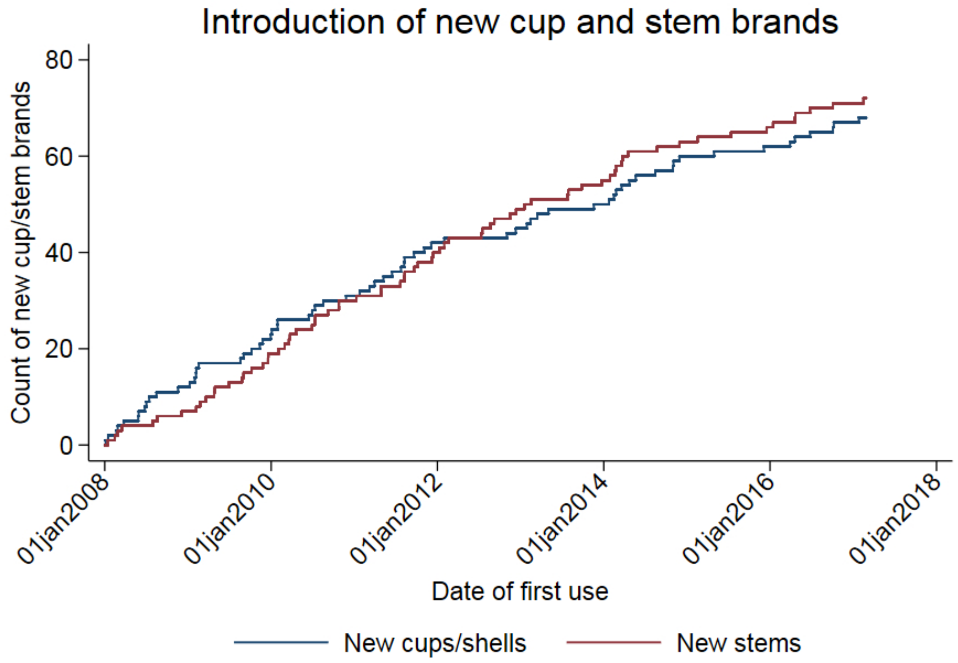

Figure S1 The cumulative introduction of new brands of cup and stem components for THRs, between January 1st 2008 and 26th February 2017

319x232mm (72 x 72 DPI)

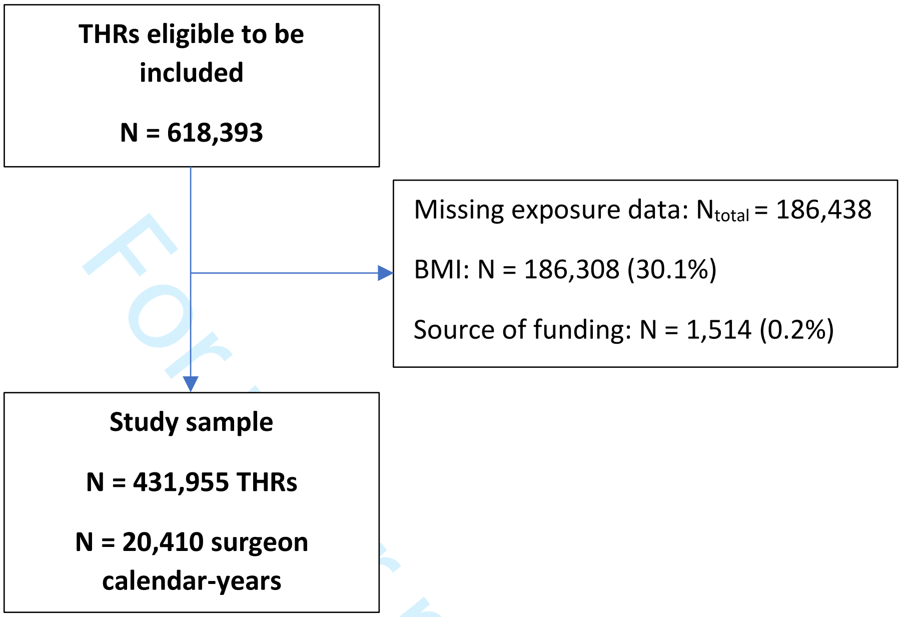

For peer review only

Reporting checklist for cohort study.

Based on the STROBE cohort guidelines.

Instructions to authors

Complete this checklist by entering the page numbers from your manuscript where readers will find each of the items listed below.

Your article may not currently address all the items on the checklist. Please modify your text to include the missing information. If you are certain that an item does not apply, please write "n/a" and provide a short explanation.

Upload your completed checklist as an extra file when you submit to a journal.

In your methods section, say that you used the STROBE cohort reporting guidelines, and cite them as:

von Elm E, Altman DG, Egger M, Pocock SJ, Gotzsche PC, Vandenbroucke JP. The Strengthening the Reporting of Observational Studies in Epidemiology (STROBE) Statement: guidelines for reporting observational studies.

		Reporting Item	Page Number
Title	#1a	Indicate the study's design with a commonly used term in the title or the abstract	1
Abstract	#1b	Provide in the abstract an informative and balanced summary of what was done and what was found	4

For peer review only

Background / rationale	#2	Explain the scientific background and rationale for the investigation being reported	6
Objectives	#3	State specific objectives, including any prespecified hypotheses	6
Study design	#4	Present key elements of study design early in the paper	7
Setting	#5	Describe the setting, locations, and relevant dates, including periods of recruitment, exposure, follow-up, and data collection	7
Eligibility criteria	#6a	Give the eligibility criteria, and the sources and methods of selection of participants. Describe methods of follow-up.	7
	#6b	For matched studies, give matching criteria and number of exposed and unexposed	See note 1
Variables	#7	Clearly define all outcomes, exposures, predictors, potential confounders, and effect modifiers. Give diagnostic criteria, if applicable	7-8
Data sources / measurement	#8	For each variable of interest give sources of data and details of methods of assessment (measurement). Describe comparability of assessment methods if there is more than one group. Give information separately for for exposed and unexposed groups if applicable.	7-8
Bias	#9	Describe any efforts to address potential sources of bias	8-9
Study size	#10	Explain how the study size was arrived at	See note 2

For peer review only

Quantitative variables	#11	Explain how quantitative variables were handled in the analyses. If applicable, describe which groupings were chosen, and why	8-9
Statistical methods	#12a	Describe all statistical methods, including those used to control for confounding	8-9
	#12b	Describe any methods used to examine subgroups and interactions	8-9
	#12c	Explain how missing data were addressed	8
	#12d	If applicable, explain how loss to follow-up was addressed	See note 3
	#12e	Describe any sensitivity analyses	8-9
Participants	#13a	Report numbers of individuals at each stage of study—eg numbers potentially eligible, examined for eligibility, confirmed eligible, included in the study, completing follow-up, and analysed. Give information separately for for exposed and unexposed groups if applicable.	9-10
	#13b	Give reasons for non-participation at each stage	9-10
	#13c	Consider use of a flow diagram	Figure S2
Descriptive data	#14a	Give characteristics of study participants (eg demographic, clinical, social) and information on exposures and potential confounders. Give information separately for exposed and	9-10

For peer review only

		unexposed groups if applicable.	
	#14b	Indicate number of participants with missing data for each variable of interest	Figure S2
	#14c	Summarise follow-up time (eg, average and total amount)	See note 5
Outcome data	#15	Report numbers of outcome events or summary measures over time. Give information separately for exposed and unexposed groups if applicable.	See note 6
Main results	#16a	Give unadjusted estimates and, if applicable, confounder-adjusted estimates and their precision (eg, 95% confidence interval). Make clear which confounders were adjusted for and why they were included	See note 7
	#16b	Report category boundaries when continuous variables were categorized	See note 8
	#16c	If relevant, consider translating estimates of relative risk into absolute risk for a meaningful time period	See note 9
Other analyses	#17	Report other analyses done—e.g., analyses of subgroups and interactions, and sensitivity analyses	9-12
Key results	#18	Summarise key results with reference to study objectives	13
Limitations	#19	Discuss limitations of the study, taking into account sources of potential bias or imprecision. Discuss both direction and magnitude of any potential bias.	13

For peer review only

Interpretation	#20	Give a cautious overall interpretation considering objectives, limitations, multiplicity of analyses, results from similar studies, and other relevant evidence.	13-15
Generalisability	#21	Discuss the generalisability (external validity) of the study results	15
Funding	#22	Give the source of funding and the role of the funders for the present study and, if applicable, for the original study on which the present article is based	3

For peer review only

Author notes

1. n/a - not relevant
2. n/a - not relevant
3. n/a - not relevant
4. 10 and Fig S2
5. n/a - not relevant
6. Tables 1 & 2
7. Tables S6 & S7
8. n/a - not needed
9. n/a - not needed

The STROBE checklist is distributed under the terms of the Creative Commons Attribution License CC-BY. This checklist was completed on 31. January 2019 using https://www.goodreports.org/, a tool made by the EQUATOR Network in collaboration with Penelope.ai
